# Alcohol Self‐Aggregation: the Preferred Configurations of the Ethanol Trimer

**DOI:** 10.1002/anie.202415229

**Published:** 2024-12-09

**Authors:** S. Indira Murugachandran, Isabel Peña, Al Mokhtar Lamsabhi, Manuel Yáñez, M. Eugenia Sanz

**Affiliations:** ^1^ Department of Chemistry King's College London London SE1 1DB United Kingdom; ^2^ Present address: Departamento de Química Física y Química Inorgánica Facultad de Ciencias-I.U. CINQUIMA Universidad de Valladolid Paseo de Belén 7 Valladolid 47011 Spain; ^3^ Departamento de Química & Institute for Advanced Research in Chemical Sciences (IAdChem) Universidad Autónoma de Madrid Cantoblanco 28049 Madrid Spain

**Keywords:** computational chemistry, clusters, H⋯ H dispersion interactions, hydrogen bonding, rotational spectroscopy

## Abstract

An atomic‐level knowledge of the aggregation of archetypal molecular systems is essential to accurately model supramolecular structures and the transition from gas to liquid phase. The structures and forces involved in ethanol aggregation have been investigated using microwave spectroscopy and extensive quantum chemical calculations. Four isomers of the ethanol trimer have been observed and identified based on comparisons between experimental and predicted spectroscopic parameters, and considering collisional relaxation in the supersonic expansion. All observed isomers exhibit O−H⋯ O hydrogen bonds between the hydroxyl groups forming a six‐membered ring. Additionally, secondary C−H⋯ O hydrogen bonds and H⋯ H dispersion contacts participate in the stabilization of the complexes with remarkably similar energy contributions. Structures where there is a mixture of *gauche* and *trans* conformations of ethanol are favored, with *gauche* conformations being predominant and no evidence of homochirality synchronization. Our results underscore the critical changes involved in aggregation as the size of the system increases and shed light on the unique properties and behavior of ethanol in chemical and biological systems.

## Graphical abstract

Four isomers of the ethanol trimer were identified by their rotational spectra aided by extensive quantum chemical calculations. All aggregates show cyclic structures linking the −OH groups through O−H⋅⋅⋅O hydrogen bonds, and additional C−H⋅⋅⋅O and H⋅⋅⋅H interactions. *Gauche* ethanol conformations are prevalent but there is no homochirality synchronization. Our results underscore the critical changes involved in alcohol aggregation as size increases.

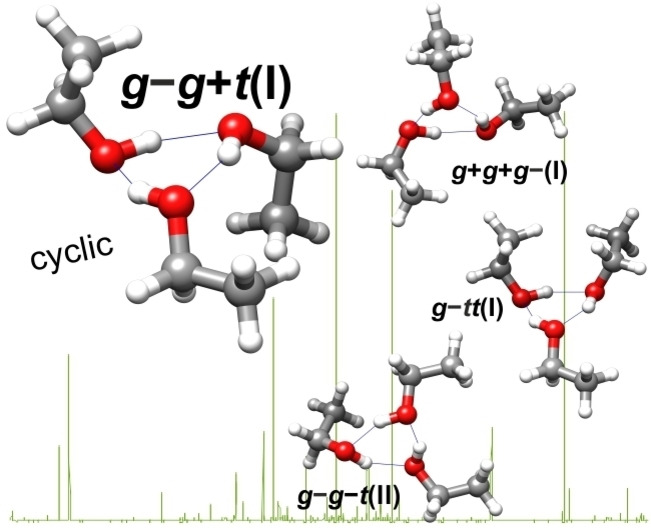

## Introduction

The study of the conformations and interactions of model molecular clusters is crucial for understanding aggregation as well as the role of clusters in chemical and biological processes, for example as pre‐reactive intermediates. Aggregation is driven by a fine balance of intra‐ and intermolecular non‐covalent interactions, and, even for small clusters, its accurate description by quantum chemical methods is a formidable challenge. Investigations in a collision‐free environment in the gas phase have the advantage of allowing identification of the relevant non‐covalent interactions without interference from the medium, and of providing direct appraisal of results from computational calculations. Gas‐phase data can also serve as a structural basis to describe the behavior in the liquid phase, for example using the quantum cluster model, and as a starting point to model liquid dynamics.[^1^, ^2^, ^3^] Experimental studies of clusters in the gas phase have greatly benefitted from the use of supersonic expansions, where weakly‐bound species are produced at the onset by collisions and survive for interrogation with spectroscopic methods.[^4^, ^5^] In this context, rotational spectroscopy, with its unrivalled resolution and incomparable sensitivity to molecular structure, is a powerful technique to identify different cluster configurations and the forces driving aggregation.[^6^, ^7^, ^8^, ^9^]

Considering small model systems, water clusters have been the most extensively studied, with investigations in the gas phase reported up to the hexadecamer.[^10^, ^11^, ^12^, ^13^, ^14^] These studies have given incredible insight into the process of water aggregation, unveiling the favored configurations of water molecules and examining O−H⋯ O hydrogen bond networks and their changes upon cluster growth. Alcohol clusters, where one of the hydrogen atoms of water is replaced by an alkyl or aryl group, exhibit a much more complex interaction network and configuration manifold. In addition to O−H⋯ O hydrogen bonding, C−H⋯ O, C−H⋯ π and dispersion interactions can occur. Moreover, conformational flexibility gives rise to many possible cluster configurations and can substantially complicate sampling of the potential energy surface. Presumably because of this, significantly fewer studies of alcohol clusters have been reported in comparison with water ones.

Ethanol, CH_3_CH_2_OH, is one of the simplest aliphatic alcohols, and probably the most important one owing to its industrial and chemical applications. Ethanol is classified as a green solvent as it is environmentally friendly, and it can also be produced from renewable sources, thus being labelled bioethanol. It is used as a biofuel and in industrial processes involving supercritical fluids.[^15^, ^16^] Owing to its amphiphilic nature, it is also considered a universal solvent, since it can interact with polar compounds through its hydrophilic hydroxyl functional group and with nonpolar compounds through its alkyl tail. This versatility, in addition to its large limits for exposure/ingestion, makes it a common and attractive choice in pharmaceutical drug production.^[17]^ Ethanol is a flexible molecule that can adopt three possible conformations, namely *trans* (*t*), *gauche*− (*g*−), and *gauche*+ (*g*+), corresponding to values of the ∠ CCOH dihedral angle of 180°, −60°, and +60°, respectively. The *trans* conformation is the global energy minimum.^[18]^ The *gauche* conformations are approximately 0.5 kJ mol^−1^ higher in energy than the *trans*, and they are equivalent enantiomers that can interconvert through quantum tunnelling.^[18]^ However, upon complexation with a chiral or prochiral molecule, the tunnelling motion of the *gauche* conformers is hindered, and they survive the duration of the spectroscopic interrogation. Therefore, all three conformations need to be considered in ethanol aggregates.

The first mention to ethanol aggregates appeared in 1936, when a signal in the IR spectrum of ethanol was attributed to them.^[19]^ Initially, ethanol dimer was thought to be cyclic, but no plausible structures were proposed.[^20^, ^21^] Later, experimental and computational studies showed that the dimer was linear, and provided evidence of the presence of multiple structures formed from the combinations of the different conformations of ethanol, giving the families *tt*, *tg*+, *g*+*t*, *g*−*t*, *g*+*g*+, *g*+*g*−, *g*−*g*+ and *g*−*g*−.[^22^, ^23^, ^24^, ^25^] However, it was not until recently that the various low‐energy isomers of ethanol dimer as well as the global minimum were identified unambiguously by rotational spectroscopy.[^26^, ^27^, ^28^] The global minimum belongs to the *g*+*g*+ family, and constitutes a good example of chirality synchronization,^[29]^ where chiral molecules adjust to each other's chirality in a concerted manner.

The ethanol trimer has also been the subject of numerous experimental and theoretical studies. Similarly to the dimer, several families can be considered attending to the conformations of the monomer, namely *g+g+g*−, *g*−*g*−*t*, *g*−*tt*, *g*−*g+t, ttt* and *g+g+g+*. It is worth mentioning that every family with a *gauche* moiety has a corresponding enantiomeric counterpart with the opposite *gauche* conformation. A vibrational spectroscopy study on ethanol clusters in the gas phase determined that the ethanol trimer has a so‐called cyclic arrangement, with all the hydroxyl groups forming O−H⋯ O hydrogen bonds and simultaneously acting as hydrogen bond donors and acceptors.^[23]^ Later, an IR cavity ring‐down investigation identified a broad vibrational band as arising from the trimer, and hypothesized the presence of several isomers given its width,^[22]^ but no further details on the structure of the trimers were provided. Early computational calculations were consistent with a cyclic configuration, and predicted that the lowest energy structure of the ethanol trimer has *C*_3_ symmetry and consists completely of *trans* ethanol (*ttt*).[^23^, ^25^] A preference for cyclic ethanol trimer was also predicted in the liquid phase, and experiments using FT‐IR and NMR in polar and non‐polar solvents confirm those predictions.^[30]^ In 2020 an expanded theoretical study of the potential energy surface of ethanol trimer was published by Malloum *et al*.,^[31]^ which yielded more low energy structures than previously reported, along with a new global minimum including the three different conformations of ethanol *g*+*g*−*t*. Similar results were obtained independently by Medel.^[32]^ However, an experimental spectroscopic study providing details on the preferred configurations adopted by ethanol trimer is lacking.

Here we present the investigation of the ethanol trimer in the gas phase using broadband Fourier transform microwave (FTMW) spectroscopy^[33]^ and extensive computational calculations. In the course of preparing this publication, Dutton et al. reported clusters of pure ethanol and water‐ethanol mixtures.^[34]^ Our results are not consistent with theirs. We were unable to find transitions corresponding to their reported isomers of ethanol trimer, but their limited experimental and theoretical data sets raise some questions on the analysis. We have observed four distinct isomers in the rotational spectrum, which belong to four different families. All observed isomers contain at least one ethanol moiety in a *gauche* conformation, and they are cyclic, exhibiting a six‐membered ring formed by O−H⋯ O hydrogen bonds between the hydroxyl groups of the monomers. In addition, secondary C−H⋯ O and H⋯ H non‐covalent interactions are present in all observed isomers, and they contribute similarly to isomer stabilization. The most abundant isomer belongs to the *g*+*g*−*t* family, consistent with predictions of the global energy minimum. Remarkably, it contains the three conformers of bare ethanol, and shows no homochirality synchronization.

## Results and Discussion

**Figure 1:**
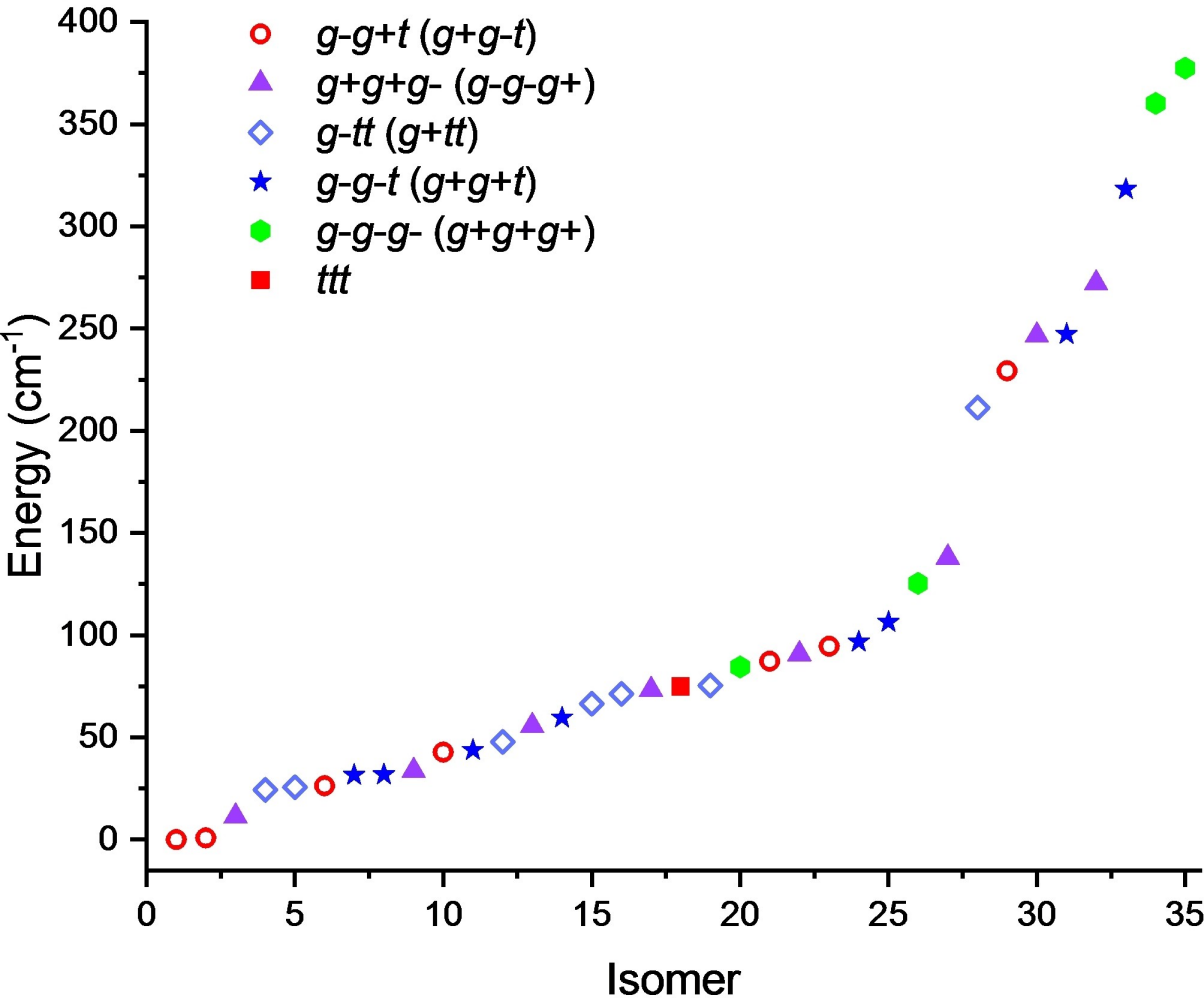
Families and zero‐point corrected relative energies of the low‐lying isomers of the ethanol trimer calculated at the B3LYP‐D3BJ/6–311++G(d,p) level of theory.

The potential energy surface (PES) of the ethanol trimer was first sampled by considering permutations of the three conformers of ethanol, and later by carrying out six separate PES searches starting from different configurations through the program CREST.^[35]^ The resulting geometries were optimized at the B3LYP‐D3BJ and MP2 levels of theory with the 6–311++G(d,p) basis set. Overall, 35 structures were predicted within 400 cm^−1^ (see Figure 1). Their rotational constants, dipole moment components and relative energies, including zero‐point and counterpoise corrections, are collected in Tables S1–S5 of the SI. All low‐energy isomers of the ethanol trimer exhibit a cyclic configuration where all hydroxyl groups are linked through O−H⋯ O bonds forming a six‐membered ring. Isomers forming a linear chain of O−H⋯ O hydrogen bonds are considerably higher in energy, with the first appearing above 850 cm^−1^. Similar results were reported in refs. [31,32], where different methods were used to generate the initial structures and perform geometry optimizations.

The isomers of the ethanol trimer can be classified into six distinct families based upon the conformation of the ethanol monomers, labelled *g+g+g*−, *g*−*g*−*t*, *g*−*tt*, *g*−*g+t, ttt* and *g+g+g+*. All complexes, except the purely *trans* (*ttt*) one, have an equivalent enantiomeric pair arising from the exchange of the *g+* and *g*− conformations. For example, a complex within the *g*−*g*−*t* family has its chiral pair in the *g+g+t* family. Differences between the structures in each family arise from changes in the ethyl tail, which can be above or below the plane of the hydrogen bonded hydroxyl groups.

The rotational spectrum of ethanol was recorded in the 2–8 GHz region using our broadband FTMW spectrometer.[^26^, ^36^] The presence of ethanol clusters was confirmed by observation of the strong transitions from ethanol dimer,[^26^, ^27^] which were removed before looking for the trimer. Extensive searches were required as our initial computational results predicted low‐energy trimer structures with considerably different spectroscopic parameters than those first identified. Eventually, four isomers of ethanol trimer were found, using the spectral simulating and fitting program PGOPHER^[37]^ along with AUTOFIT,^[38]^ the automated fitting tool for molecular spectra incorporated in PGOPHER. The measured transitions were fitted using the semi‐rigid rotor Hamiltonian of Watson in the A reduction and the *I* ^*r*^ representation,^[39]^ and the SPFIT/SPCAT^[40]^ programs. The resulting experimental spectroscopic constants are given in Table 1.

**Table 1:** Experimental spectroscopic parameters of the four observed isomers of the ethanol trimer, and their comparison with equilibrium B3LYP‐D3BJ/6–311++G(d,p) theoretical parameters (*B*_e_) and ground state theoretical parameters (*B*_0_) at the BPCS//B3LYP level of theory.^[a]^

	Isomer 1 *g*−*g+t*(I)		Isomer 2 *g+g+g*−(I)		Isomer 3 *g*−*tt*(I)		Isomer 4 *g*−*g*−*t*(II)	
	Experimental	*B_0_*/*B_e_*	Experimental	*B_0_*/*B_e_*	Experimental	*B_0_*/*B_e_*	Experimental	*B_0_*/*B_e_*
*A* ^[b]^ (MHz)	1217.4761(19)^[i]^	1205.3/1271.9	1200.01318(77)	1197.5/1233.0	1211.3135(11)	1214.7/1265.8	1086.27337(10)	1079.8/1094.4
*B* (MHz)	807.11975(49)	793.9/799.7	820.05962(57)	804.9/815.8	752.78429(53)	753.2/748.1	833.52668(61)	824.6/842.7
*C* (MHz)	565.11681(27)	554.8/572.4	571.24062(50)	564.6/575.9	514.44626(39)	515.2/522.2	535.45543(43)	531.7/542.7
*κ* ^[c]^	−0.26	−0.26/−0.35	−0.21	−0.24/−0.27	−0.32	−0.32/−0.39	0.08	0.07/0.09
*Δ_J_* (kHz)^[d]^	0.420(10)	0.32^[j]^	0.3851(91)	0.34^[j]^	0.4535(95)	0.25^[j]^	0.434(13)	1.39^[j]^
*Δ_JK_* (kHz)	0.334(36)	0.19	0.256(37)	0.22	0.783(43)	0.41	−0.432(57)	−3.37
*Δ_K_* (kHz)	1.69(38)	1.40	–	2.58	1.74(10)	1.59	3.420(90)	2.12
*δ_J_* (kHz)	0.1141(50)	0.08	0.1175(58)	0.09	0.1282(46)	0.07	0.1297(61)	0.44
*δ_K_* (kHz)	0.435(44)	0.38	–	0.33	0.978(49)	0.47	–	−2.36
|*μ_a_* |/|*μ_b_* |/|*μ_c_* |^[e]^	y/y/y	0.8/0.4/0.6	y/n/y	0.7/0.2/0.7	y/n/y	0.5/0.2/1.1	n/y/y	0.1/0.4/1.2
*σ* ^[f]^ (kHz)	4.9	–	5.1	–	4.0	–	4.5	–
*N* ^[g]^	74	–	36	–	39	–	35	–
Δ*E*_ZPC_^[h]^	–	0.0		11.4		24.4		31.6

[a] BPCS//B3LYP indicates that vibrational corrections were calculated at B3LYP‐D3BJ/6–311++G(d,p) level on the BPCS equilibrium geometries. [b] *A*, *B* and *C* are the rotational constants. [c] *κ* is Ray's asymmetry parameter. [d] *Δ_J_*, *Δ_JK_*, *Δ_K_*, *δ_J_*, and *δ_K_* are the quartic centrifugal distortion constants. [e] |*μ*_a_|, |*μ*_b_| and |*μ_c_* | are the absolute values of the B3LYP‐D3BJ/6–311++G(d,p) electric dipole moment components in Debye; *y* and *n*, yes and no, indicate whether *a‐*, *b‐* and *c‐*type transitions are observed or not. [f] *σ* is the rms deviation of the fit. [g] *N* is the number of fitted transitions. [h] Δ*E*_ZPC_ are the B3LYP−D3BJ/6–311++G(d,p) relative energies including zero‐point corrections in cm^−1^. [i] Standard error in parentheses in units of the last digit. [j] Values for the centrifugal distortion constants from harmonic vibrational calculations at the B3LYP‐D3BJ/6–311++G(d,p) level of theory on B3LYP‐D3BJ/6–311++G(d,p) geometries.

Assignment of the observed ethanol trimer isomers to specific structures was not straightforward. Typically, the first step is comparing the experimental and theoretical rotational constants to determine the predicted structures that could match observations. We also compared the experimental and predicted planar moments, which provide complementary information to rotational constants on out‐of‐plane contributions. Secondly, the predicted dipole moment components of isomers with theoretical rotational constants close to the experimental ones are examined. In our experiment, line intensity is proportional to the square of the corresponding dipole moment component, and therefore observation of *a*‐, *b*‐ and *c*‐type transitions should be consistent with predicted values of *μ_a_*, *μ_b_* and *μ_c_*. After considering these two steps, the observed isomers of ethanol trimer could match several predicted structures. Thus, we took into account the predicted relative energies and possible relaxation pathways between isomers. Conformational relaxation of higher‐energy species to lower‐energy ones is well known to occur in supersonic jets, by collisions with the carrier gas, when interconversion barriers are low (typically below 4.8 kJ mol^−1^).^[41]^ Interestingly, the calculated interconversion barriers between the isomers within each family are all consistently predicted to be below 3.6 kJ mol^−1^ (Table S9), making relaxation very likely. Considering all the above (see details in the SI) isomers 1–4 are assigned as ***g***−***g+t*** (**I**), ***g+g+g***−(**I**), ***g***−***tt*** (**I**) and ***g***−***g***−***t*** (**II**) respectively. Isomers from the *g+g+g+* and *ttt* families are higher in energy, which may explain their non‐observation. Additionally, we carried out anharmonic vibrational calculations using composite methods[^42^, ^43^] to compare theoretical ground state rotational constants with experimental ones. Their results also support our assignment (see Table 1, and Tables S10, S11).

The relative abundances of the observed complexes can be estimated considering that in our experiment they are directly proportional to line intensity and inversely proportional to the square of the corresponding dipole moment component. Since all observed isomers exhibit *c*‐type lines, we compared the intensity of common *c*‐type transitions and found that the relative abundances are ***g***−***g+t*** (**I**) : ***g***−***tt*** (**I**) : ***g+g+g***−(**I**) : ***g***−***g***−***t*** (**II**)=8.6 : 2.2 : 1.3 : 1. The most abundant isomer is ***g***−***g+t*** (**I**), although it is worth noting that the experimental abundances are likely to have contributions from relaxation from higher‐energy isomers.

**Figure 2:**
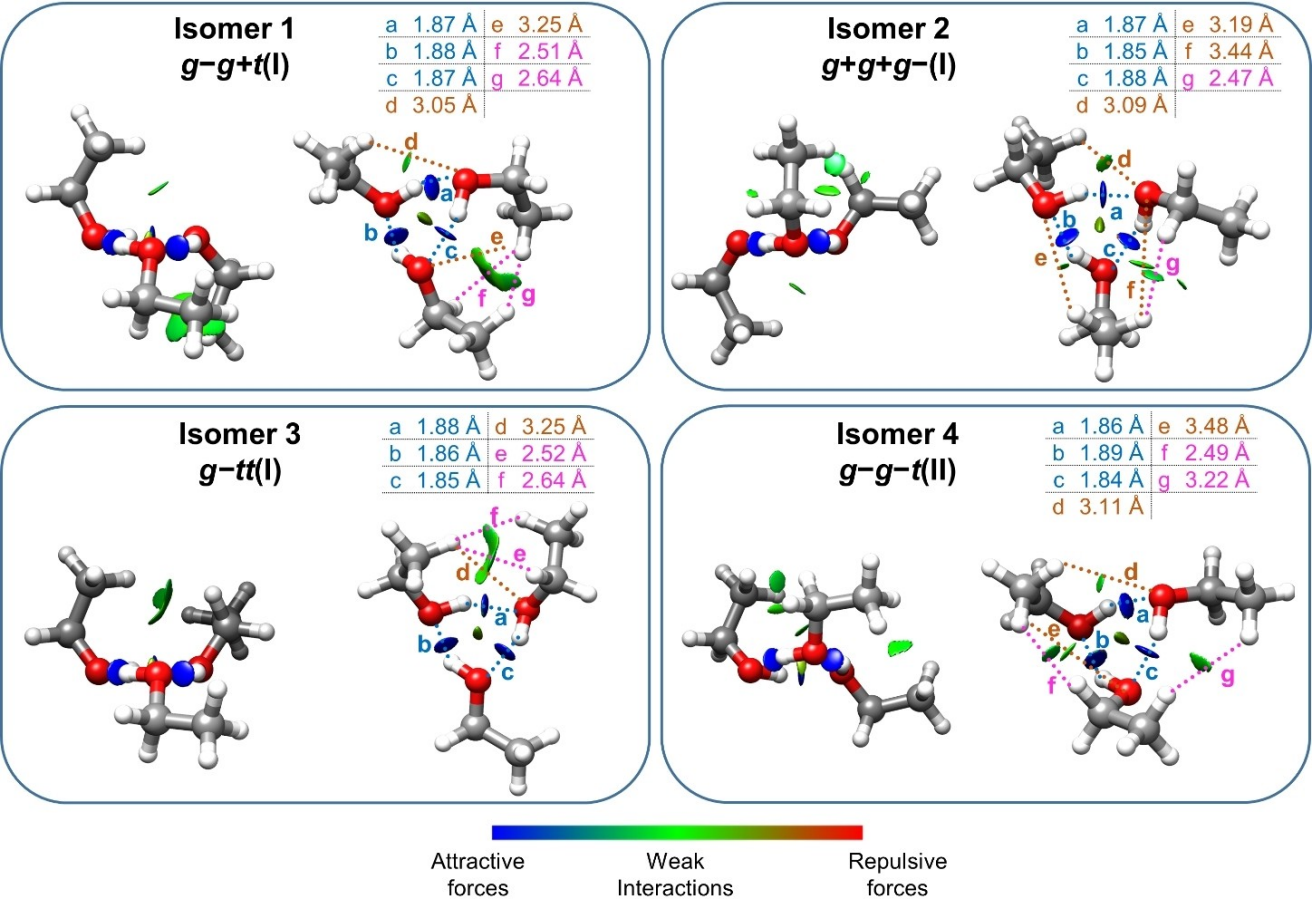
B3LYP‐D3BJ/6–311++G(d,p) structures of the observed isomers of the ethanol trimer overlaid with NCI plots *s*=0.5 for values of sign(λ2)*ρ* from −0.025 to +0.025 au. Values for O−H⋯ O bond lengths are in blue, for C−H⋯ O in brown, and for H⋯ H contacts in pink. H⋯ H distances visible in the NCI but longer than 3.0 Å are not explicitly indicated.

In all isomers the hydroxyl groups of the three ethanols form O−H⋯ O bonds that close a six‐membered planar ring. Each hydroxyl group acts simultaneously as a hydrogen bond donor and acceptor, which results in a strong cooperative network.[^44^, ^45^] In addition to O−H⋯ O bonds, all trimers show C−H⋯ O interactions and H⋯ H dispersion contacts. Attractive interactions in the observed complexes are visualized using the non‐covalent interaction (NCI) method (see Figure 2),[^46^, ^47^] which is based on the analysis of the electronic density and its derivatives. Strong attractive non‐covalent interactions such as O−H⋯ O bonds appear as blue isosurfaces, while weaker attractive non‐covalent interactions such as C−H⋯ O and H⋯ H appear as green isosurfaces. Further insight can be obtained from natural bonding orbital (NBO)[^48^, ^49^] (see Table S12) and QTAIM^[50]^ analysis (see Figure S1). The dominating interactions are the O−H⋯ O hydrogen bonds, which are very similar for all the isomers observed, with overall energy contributions of 160.3–163.6 kJ mol^−1^. Most interestingly, H⋯ H dispersion contacts clearly appear in the QTAIM graphs as bond critical points. The QTAIM graphs also show the existence of ring critical points not only associated with the six‐membered rings involving the hydroxyl groups but also with the weak interactions between the alkyl groups. This indicates that these H⋯ H interactions, despite being weak, clearly contribute to the stability of the isomers, and thus the choice of a DFT formalism including dispersion is important. The H⋯ H interactions are in the range of 0.96–1.59 kJ mol^−1^, of the same order of magnitude as the C−H⋯ O interactions, which have energy contributions between 0.71–1.30 kJ mol^−1^. However, the latter do not appear in the QTAIM graphs. It should be noted that the distances involved in the C−H⋯ O interactions are all over 3 Å, larger than the sum of the van der Waals radii of the carbon and oxygen atoms (2.72 Å),^[51]^ probably a result of maximising the O−H⋯ O interactions coupled with the constraints imposed by the geometry of ethanol.

The *gauche* conformations of ethanol seem to be preferred in the ethanol trimer. Of the observed isomers, ***g+g+g***−(**I**) is solely composed of *g+* and *g*− *gauche* conformations, while ***g***−***g+t*** (**I**) and ***g***−***g***−***t*** (**II**) involve two *gauche* conformers out of three. Only ***g***−***tt*** (**I**) presents one *gauche* and two *trans* ethanol monomers. A preference for *gauche* over *trans* conformations has been reported for complexes of ethanol where there exist non‐covalent interactions besides the hydrogen bonds formed by the hydroxyl group.[^26^, ^27^, ^52^, ^53^, ^54^, ^55^, ^56^, ^57^] These additional non‐covalent interactions compensate for the slightly higher energy of *gauche* over *trans* ethanol, making *gauche*‐containing complexes energetically favorable. Our results are consistent with this, as all observed isomers of the ethanol trimer exhibit various C−H⋯ O and H⋯ H interactions. However, it seems at odds with the non‐observation of a *g+g+g+* isomer despite repeated searches. Homochiral *g+g+g+* isomers are predicted to be higher in energy than the detected isomers. In fact, only 4 *g+g+g+* isomers are predicted within 400 cm^−1^. The lowest‐energy *g+g+g+* isomer is above the only *ttt* isomer in this energy range (see Figure 1) and displays merely one C−H⋯ O hydrogen bond besides the three O−H⋯ O bonds formed by the hydroxyl groups. We hypothesize that the reduced number of additional non‐covalent interactions results in *g+g+g+* and *ttt* isomers lying at higher energies. The *ttt* isomer within 400 cm^−1^ presents one H⋯ H contact and no C−H⋯ O interactions. Thus, in the ethanol trimer a combination of *gauche* and *trans* conformations favors the establishment of non‐covalent interactions in addition to O−H⋯ O hydrogen bonds.

The conformational preferences of the ethanol trimer differ markedly from those of the dimer,[^26^, ^28^] where the global energy minimum is the homochiral complex *g+g+*. In the ethanol trimer, isomers composed of only one type of ethanol conformers are disfavored, indicating that there is no chirality synchronization for a homochiral complex. However, the fact that the most abundant isomer belongs to the *g+g*−t family and the general preference for the inclusion of *gauche* structures in the observed isomers, imply the existence of chirality synchronisation for heterochiral complexes. If similar behavior is displayed by larger ethanol clusters, a mixture of *gauche* and *trans* conformations can be expected, without a significant proportion of homochiral clusters.

The ethyl tails of the observed isomers display an up/up/down (uud) or down/down/up (ddu) configuration with respect to the plane of the hydrogen bonded ring (see Figure 2). This preference is also displayed by the methyl tails in the methanol trimer[^58^, ^59^, ^60^] and by the H atoms not participating in hydrogen bonds in the water trimer.^[59]^ The preferred arrangements, however, depend on a very fine balance of non‐covalent interactions. For example, in phenol^[6]^ and 2,2,2‐trifluoroethanol trimers,^[7]^ the configurations of the tails change to uuu/ddd. In the phenol trimer,^[6]^ this enables C−H⋯ π bonds between the benzene rings, while additional C−H⋯ F and C−F⋯ F−C interactions are established in 2,2,2‐trifluoroethanol trimer.^[7]^ In the ethanol trimer, the uuu or ddd configurations are predicted higher in energy, above 2.4 kJ mol^−1^ at B3LYP‐D3BJ and MP2 levels of theory than the uud/ddu ones.

The cyclic O−H⋯ O hydrogen bond arrangement of the ethanol trimer has also been observed in the water trimer,^[59]^ and in the alcohol trimers of methanol,^[44]^ phenol^[6]^ and 2,2,2‐trifluoroethanol.^[7]^ In fact, the hydrogen bonding networks of the aforementioned trimers hardly vary as alcohol tails increase their size and change from aliphatic to aromatic, attending to predicted values for the O⋯ O distances by B3LYP‐D3BJ and MP2 calculations (see Table S15). Experimental values of the O⋯ O distances of phenol and water trimers are reported as 2.760(70) Å^[6]^ and 2.88 Å,^[61]^ respectively.

The cyclic O−H⋯ O arrangement, with a continuous chain of hydrogen bond donors and acceptors, reinforces hydrogen bonds and results in cooperativity effects,[^44^, ^45^] making these isomers preferred over those forming linear chains of hydrogen bonds overcoming ring strain. We have investigated whether the observed gas‐phase cyclic isomers remain significantly stable when moving to the liquid phase, where other interactions will be in competition. We carried out similar conformational sampling as the one performed in the gas phase but in ethanol solution using a polarizable continuum model^[62]^ (see details in SI). Our scrutiny found that in solution, the most stable isomer is not cyclic (see Table S16), and so are the next isomers in energy. Cyclic isomers are still local minima in solution, but since they are more compact, the interaction with the solvent is less efficient and they lay higher in energy. The first cyclic structure is ***g***−***tt*** (**VII**), a uuu configuration, lying 3.5 kJ mol^−1^ above the global minimum. In comparison, ***g***−***tt*** (**VII**) lies at 2.5 kJ mol^−1^ in the gas phase. Another interesting result is that *gauche* ethanol is more predominant in the liquid phase, with all low‐energy acyclic structures involving two or three *gauche* ethanols.

## Conclusions

In summary, four unique isomers of ethanol trimer have been identified from the analysis of the rotational spectrum. All complexes have a cyclic ring formed by O−H⋯ O bonds involving the three hydroxyl groups, which constitute the largest stabilizing contributions to the structure. Secondary C−H⋯ O hydrogen bonds and H⋯ H contacts are also present in all isomers and show remarkably similar energy contributions to one another. The observed clusters display a mixture of ethanol conformations where *gauche* forms are predominant but there is no homochirality synchronization. Isomers composed of only one type of ethanol conformer are disfavored.

The observed structural motifs and interaction patterns of ethanol trimer provide insight into the gas‐phase aggregation of an archetypal alcohol, and can serve as benchmark for the development of theoretical models and simulations by the wider chemistry community. It will aid in the accurate prediction of properties and behavior of larger molecular assemblies, and to understand aggregation processes in different media.

## Supporting Information

Supporting Information includes details on the computational and experimental methods, assignment, tables of spectroscopic constants, NCI plots, and lists of measured transitions. The authors have cited additional references within the Supporting Information.

## Conflict of Interests

The authors declare no conflict of interest.

1

## Supporting information

As a service to our authors and readers, this journal provides supporting information supplied by the authors. Such materials are peer reviewed and may be re‐organized for online delivery, but are not copy‐edited or typeset. Technical support issues arising from supporting information (other than missing files) should be addressed to the authors.

Supporting Information

## Data Availability

The data that support the findings of this study are available in the supplementary material of this article.
