# Supplementary material for: Alcohol Self‐Aggregation: the Preferred Configurations of the Ethanol Trimer
Source: Angew Chem Int Ed Engl. 2024 Dec 9;64(12):e202415229. doi: 10.1002/anie.202415229 (PMC11914937; doi:10.1002/anie.202415229)
Supplement: Supplementary file 1 — Supporting Information [file ANIE-64-e202415229-s001.pdf]

Angewandte Chemie
Eine Zeitschrift der Gesellschaft Deutscher Chemiker

Supporting Information

Alcohol Self-Aggregation: the Preferred Configurations of the Ethanol Trimer

*S. Indira Murugachandran, I. Peña, A. Mokhtar Lamsabhi, M. Yáñez, M. Eugenia Sanz**

Supporting Information
©Wiley-VCH 2021
69451 Weinheim, Germany

Alcohol Self-Aggregation: the Preferred Configurations of the Ethanol Trimer

S. Indira Murugachandran,[a] Isabel Peña,[a],† Al Mokhtar Lamsabhi,[b] Manuel Yáñez,[b] M. Eugenia Sanz*[a]

[a] Dr. S. I. Murugachandran, Dr. I. Peña, Dr. M. E. Sanz
Department of Chemistry
King's College London
London, SE1 1DB, United Kingdom
E-mail: maria.sanz@kcl.ac.uk

[b] Prof. A. M. Lamsabhi, Prof. Dr. M. Yáñez
Departamento de Química & Institute for Advanced Research in Chemical Sciences (IAdChem)
Universidad Autónoma de Madrid
Cantoblanco, 28049, Madrid, Spain

† Present address: Departamento de Química Física y Química Inorgánica, Universidad de Valladolid, Paseo de Belén 7, Valladolid 47011, Spain.

Supporting information for this article is given via a link at the end of the document.

DOI: 10.1002/anie.2021XXXXX

Table of Contents

S. 1

S1. Experimental methods.......... 4

S2. Computational Details 4

Table S1. Calculated spectroscopic parameters for the *g−tt* (*g+tt*) isomers at the MP2/6-311++G(d,p) and B3LYP-D3BJ/6-311++G(d,p) levels of theory. 6

Table S2. Calculated spectroscopic parameters for the *g−g+t* (*g+g−t*) isomers at the MP2/6-311++G(d,p) and B3LYP-D3BJ/6-311++G(d,p) levels of theory. 7

Table S3. Calculated spectroscopic parameters for the *g−g−t* (*g+g+t*) isomers at the MP2/6-311++G(d,p) and B3LYP-D3BJ/6-311++G(d,p) levels of theory. 8

Table S4. Calculated spectroscopic parameters for the *g+g+g−* (*g−g−g+*) isomers at the MP2/6-311++G(d,p) and B3LYP-D3BJ/6-311++G(d,p) levels of theory. 9

Table S5. Calculated spectroscopic parameters for the homochiral *g+g+g+*(*g−g−g−*) and *ttt* ethanol trimer isomers at the MP2/6-311++G(d,p) and B3LYP-D3BJ/6-311++G(d,p) levels of theory. 10

S3. Assignment 11

Table S6. Comparison of the experimental and theoretical planar moments for the potential assignments of each isomer of ethanol trimer observed in this study. 11

Table S7. Experimental spectroscopic parameters of the observed 1-^{13}C isotopologues of isomer 1 of ethanol trimer. 1-^{13}C labelling is given in Figure S2. 13

Table S8. Experimental coordinates of the 1-^{13}C atoms for isomer 1 of ethanol trimer compared with equilibrium coordinates from possible structures from B3LYP-D3BJ and MP2 calculations with the 6-311++G(d,p) basis set. 1-^{13}C labelling is given in Figure S2. 13

Table S9. Interconversion barrier heights calculated at the B3LYP-D3BJ/6-311++G(d,p) level of theory. 14

Table S10. Comparison of the theoretical equilibrium and ground state rotational constants (in MHz) with the experimental values for the isomers of the ethanol trimer. The equilibrium constants are at the B3LYP/6-311+G(d,p) level of theory and the vibrational corrections were calculated at the B3LYP/6-311++G(d,p) level on the BPCS-corrected equilibrium structures. 16

Table S11. Comparison of the theoretical equilibrium and ground state rotational constants (in MHz) with the experimental values for the isomers of the ethanol trimer. The equilibrium constants are at the rDSD/cc-pVTZ-F12 level of theory and the vibrational correction at B3LYP/6-311+G(d,p) level. 17

S4. Natural Bonding Orbital Data 18

Table S12. Intermolecular stabilising energy contributions (≥0.42 kJ mol^{-1}) for isomers 1 – 4 of ethanol trimer from Natural Bond Orbital (NBO) analysis at the B3LYP-D3BJ/6-311++G(d,p) level of theory. 18

Table S13. Intermolecular stabilising energy contributions for the H···H contacts of isomers 1 – 4 of ethanol trimer from Natural Bond Orbital (NBO) analysis at the B3LYP-D3BJ/6-311++G(d,p) level of theory. 20

S5. QTAIM Analysis 21

Figure S1. Results form the QTAIM analysis of the observed ethanol trimer isomers. Bond paths are shown in orange. Critical Points (CPs) on bond paths are bond critical points (BCPs), shown with yellow spheres and the corresponding electron densities (in a.u.) of BCPs are given. CPs not on bond paths are ring critical points (RCPs), shown with green spheres. 21

Table S14. Calculated O···O distances at B3LYP-D3BJ/6-311++G(d,p) and MP2/6-311++G(d,p) (given in brackets) levels of theory. Oxygen nomenclature is given in Figure S2. 22

Table S15. Calculated O···O distances of several alcohol trimers and the water trimer at B3LYP-D3BJ/6-311++G(d,p) and MP2/6-311++G(d,p) levels of theory. 22

Figure S2. Oxygen labelling for the calculated O···O distances and labelling for the 1-^{13}C carbon isotopologues. 22

S6. Liquid Phase Results........ 23

Table S16. Calculated low-energy liquid phase structures up to 300 cm^{-1} from B3LYP-D3BJ/6-311++G(d,p) level of theory with the polarizable continuum model SMD using ethanol as a solvent. Structures named CONFX are from the CREST-CENSO calculation described in section S2, where X corresponds to the number of the output. 23

S7. Measured frequencies 28

Table S17. Measured frequencies and residuals (in MHz) of the rotational transitions of the parent species of *g−g+t*(I)........ 28

Table S18. Measured frequencies and residuals (in MHz) of the rotational transitions of first 1-^{13}C isotopologue of *g−g+t*(I). 1-^{13}C labelling is given in Figure S2........ 29

Table S19. Measured frequencies and residuals (in MHz) of the rotational transitions of second 1-^{13}C isotopologue of *g−g+t*(I). 1-^{13}C labelling is given in Figure S2........ 30

Table S20. Measured frequencies and residuals (in MHz) of the rotational transitions of third 1-^{13}C isotopologue of *g−g+t*(I). 1-^{13}C labelling is given in Figure S2........ 30

Table S21. Measured frequencies and residuals (in MHz) of the rotational transitions of the parent species of *g+g+g−*(I). 31

Table S22. Measured frequencies and residuals (in MHz) of the rotational transitions of the parent species of *g−tt*(I). 32

Table S23. Measured frequencies and residuals (in MHz) of the rotational transitions of the parent species of *g−g−t*(II)........ 33

Table S24. Cartesian coordinates of isomer *g-g+t*(I) from B3LYP-D3BJ/6-311++G(d,p) calculations. 34

Table S25. Cartesian coordinates of isomer *g+g+g−*(I) from B3LYP-D3BJ/6-311++G(d,p) calculations. 35

Table S26. Cartesian coordinates of isomer *g−tt*(I) from B3LYP-D3BJ/6-311++G(d,p) calculations. 36

Table S27. Cartesian coordinates of isomer *g−g−t*(II) from B3LYP-D3BJ/6-311++G(d,p) calculations. 37

S8. References 38

S9. Author Contributions........ 38

S1. Experimental methods

The microwave spectrum of ethanol was collected in the 2-8 GHz range on the CP-FTMW spectrometer located at King's College London. The setup of this instrument has been described previously.[1,2] Absolute ethanol (99%) and 1-^{13}C ethanol (98%) were purchased and used without further purification. They were placed in an external reservoir and seeded in neon at 5 bar. Due to ethanol's high vapour pressure we used an injection system with a needle valve to reduce the amount of ethanol seeded in the neon carrier gas.

The mixture was introduced into our vacuum chamber through a 1 mm diameter nozzle. The collisions occurring at the start of the supersonic expansion caused the formation of ethanol complexes which then interacted with four chirped microwave pulses. These pulses were 4 µs long and varied linearly in the 2-8 GHz frequency range, with a spacing of 30 µs. The microwave pulses were broadcast into the chamber via a microwave horn. After each excitation pulse, molecular emission signals were collected for 20 µs using a second microwave horn, amplified, and then stored in the time domain. The final spectra were obtained by using a fast Fourier transform to convert 2.5 M free induction decays (FIDs) from the time domain into the frequency domain. The spectrum of isotopically enriched 1-^{13}C-ethanol: ethanol (1:2 mixture, 4.9 million FIDs) was recorded in a similar manner in order to determine the substitution position of the alpha carbon (the carbon adjacent to the hydroxyl group) of each ethanol in the complexes.

S2. Computational Details

S2.1. Potential energy surface sampling and geometry optimisation

To aid assignment of the experimental spectrum it is important to thoroughly explore the potential energy surface (PES) to predict all possible structures of the complex and to determine which ones are lower in energy. Ethanol trimer was studied through various computational methods. A first exploration of the PES considered 27 possible permutations of the ethanol trimer complex with the *g+*, *g−* and *t* configurations of ethanol. These generated structures were optimised at B3LYP-D3BJ[3–6] (with the ultrafine grid) and MP2 levels of theory with the 6-311++G(d,p) basis set using the Gaussian09[7] suite of programs. Their relative energies span 22 kJ mol^{-1}. Of these, 10 structures were predicted within 5.5 kJ mol^{-1}.

Later we used CREST[8], a conformational search program, to perform an additional search taking the lowest energy from the first PES exploration as starting structure. This CREST search produced 126 possible isomers, which were further optimised with B3LYP-D3BJ/6-311++G(d,p) yielding 20 new structures below 5.5 kJ mol^{-1}. This indicated that the PES of ethanol trimer was surprisingly complex and flat, and needed further exploration. Hence four more searches with various starting configurations of ethanol trimer were performed using CREST. Of these, two returned new structures below 5.5 kJ mol^{-1}. In parallel to our investigation, a paper was published where the configurations of ethanol clusters of various sizes were predicted theoretically[9] Because they used different computational approaches from us, we also optimised the ethanol trimer structures given in ref.[9] using B3LYP-D3BJ/6-311++G(d,p). This yielded one additional structure below 5.5 kJ mol^{-1} that was not predicted by our six different conformational searches.

In total we predicted 35 structures below 5.5 kJ mol^{-1} at B3LYP-D3BJ/6-311++G(d,p) level of theory, which are given in Table S1-S5 along with their predicted spectroscopic parameters and relative energies, as well as their zero-point and counterpoise corrected energies.[10] Of these, 12 are new structures separate from those reported in ref.[9]

The complexes can be divided into six distinct families according to their composing monomers, namely *g+g+g−*, *g−g−t*, *g−tt*, *−g+t*, *ttt* and *g+g+g+*. The *g+* and *g−* conformations are prochiral, they are undistinguishable enantiomers for the monomer but upon complexation with another chiral or prochiral molecule they can be distinguished. As a result, all complexes, except the purely trans (*ttt*) ethanol trimers, have a chiral pair which is

degenerate. For example, for the purely homochiral trimers *g+g+g+* the chiral pair will be *g−g−g−*; for *g−g−t* it will be *g+g+t*.

S2.2. Anharmonic vibrational calculations

We carried out anharmonic vibrational frequency calculations on the ethanol trimer structures that could be possible matches for the experimentally observed species to obtain the theoretical ground state rotational constants and compare them with the experimental ones. We used two different approaches:

1. The first approach is based on the bond-corrected Pisa composite (BPCS) scheme[11]. We applied it to the structures previously optimized at our computational level, obtaining the BPCS equilibrium geometries, and then vibrational corrections were obtained and subsequently incorporated into the rotational constants. This procedure is based on the following equation:

   $B_0 = B_{eq}$(B3LYP-D3BJ/6-311++G(d,p)) + ΔB(BPCS//B3LYP)

   Here the vibrational correction ΔB(BPCS//B3LYP) is calculated on the BPCS equilibrium geometries using the B3LYP-D3BJ/6-311++G(d,p) method within the VT2 approach in Gaussian. The results are listed in Table S10.

2. The second approach follows a similar approach as the calculations on ethanolamine-$(H_2O)_{1\text{-}7}$ [12], considering the equilibrium geometries optimised at the revDSD-PBEP86-D3BJ/cc-pVTZ-F12 level of theory (and the vibtational corrections at the B3LYP-D3BJ/6-311++G(d,p) level. The results are listed in Table S11.

   $B_0 = B_{eq}$(rDSD/cc-pVTZ-F12) + ΔB(B3LYP)

S2.3. Liquid phase calculations

For the liquid phase calculations, an additional CREST calculation was performed using isomer 1 as the starting structure. The obtained ensamble was sorted via the CENSO[13] algorithm utilising the low cost PBEh-3c/def2-mSVP[14,15] functional in the optimization part. The complexes were simulated in solution phase with ethanol as a solvent. The CREST-CENSO calculation output and all structures given in tables S1-5 were then optimised at the B3LYP-D3BJ/6-311++G(d,p) level of theory with the polarisable continuum model SMD[16] using ethanol as a solvent. Results are given in table S15.

Table S1. Calculated spectroscopic parameters for the *g−tt* (*g+tt*) isomers at the MP2/6-311++G(d,p) and B3LYP-D3BJ/6-311++G(d,p) levels of theory.

	***g−tt*(I)**		***g−tt*(II)**		***g−tt*(III)**		***g−tt*(IV)**	
	**B3LYP**	**MP2**	**B3LYP**	**MP2**	**B3LYP**	**MP2**	**B3LYP**	**MP2**
**A^a (MHz)**	1265.8	1229.5	1082.8	1095.3	1034.1	1054.0	1327.3	1306.4
**B (MHz)**	748.1	760.8	821.4	811.3	861.7	849.1	685.8	688.1
**C (MHz)**	522.2	521.1	525.4	526.3	525.3	525.8	475.1	476.7
**κ^b**	-0.39	-0.32	0.06	0.00	0.32	0.22	-0.51	-0.49
**$\|\mu_a\|^c$(D)**	0.5	0.4	0.4	0.5	0.5	0.5	0.4	0.4
**$\|\mu_b\|$ (D)**	0.2	0.1	0.2	0.1	0.5	0.6	0.1	0.0
**$\|\mu_c\|$ (D)**	1.1	1.1	1.1	1.0	0.7	0.7	1.0	0.8
**ΔE^d (cm^{-1})**	59.0	90.4	73.6	64.7	108.5	89.8	144.8	130.1
**ΔE_{ZPC}^e (cm^{-1})**	24.4	36.2	25.7	12.3	47.8	29.0	66.5	68.0
**ΔE_{CP}^f (cm^{-1})**	82.7	124.4	111.7	118.7	137.7	135.0	170.1	131.1
Top View	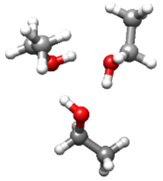		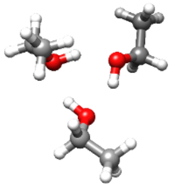		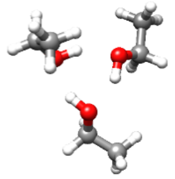		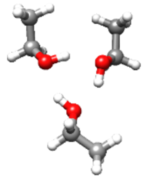	

	***g−tt*(V)**		***g−tt*(VI)**		***g−tt*(VII)**	
	**B3LYP**	**MP2**	**B3LYP**	**MP2**	**B3LYP**	**MP2**
**A^a (MHz)**	1215.8	1271.1	1135.3	1180.7	1154.4	1118.2
**B (MHz)**	736.2	738.1	740.0	724.0	885.2	885.4
**C (MHz)**	486.6	500.0	474.1	475.3	613.7	599.7
**κ^b**	-0.32	-0.38	-0.20	-0.30	0.00	0.10
**$\|\mu_a\|^c$(D)**	0.1	0.0	0.1	0.3	0.3	0.3
**$\|\mu_b\|$ (D)**	0.4	0.4	0.3	0.2	0.0	0.0
**$\|\mu_c\|$ (D)**	1.2	1.3	1.0	0.8	3.3	3.2
**ΔE^d (cm^{-1})**	147.1	93.3	153.7	116.6	270.8	300.7
**ΔE_{ZPC}^e (cm^{-1})**	71.3	54.2	75.5	62.6	211.4	199.7
**ΔE_{CP}^f (cm^{-1})**	174.9	144.9	176.0	98.9	321.4	403.6
Top View	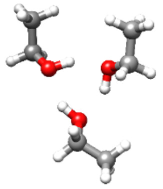		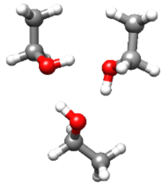		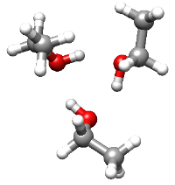	

[a] *A, B* and *C* are the rotational constants; [b] κ is Ray's asymmetry parameter; [c] $|\mu_a|$, $|\mu_b|$ and $|\mu_c|$ are the absolute values of the electric dipole moment components along the principal inertial axes; [d]ΔE are the relative energies; [e] ΔE_{ZPC} are the relative energies including zero-point corrections; [f] ΔE_{CP} are the counterpoise corrected energies.

Table S2. Calculated spectroscopic parameters for the *g−g+t* (*g+g−t*) isomers at the MP2/6-311++G(d,p) and B3LYP-D3BJ/6-311++G(d,p) levels of theory.

	g−g+t(I) *[g]		*g−g+t*(II) *		*g−g+t*(V)		*g−g+t*(I)	
	**B3LYP**	**MP2**	**B3LYP**	**MP2**	**B3LYP**	**MP2**	**B3LYP**	**MP2**
A[a] **(MHz)**	1271.9	1253.3	980.1	989.4	1384.6	1365.5	1125.3	1168.3
B **(MHz)**	799.7	800.0	971.1	955.3	712.3	712.6	825.0	808.1
C **(MHz)**	572.4	569.5	571.4	573.9	523.9	523.8	529.7	525.6
**κ**[b]	-0.35	-0.33	0.96	0.84	-0.56	-0.55	-0.01	-0.12
$\|\mu_a\|$[c]**(D)**	0.8	0.7	0.5	0.1	0.3	0.3	0.4	0.4
$\|\mu_b\|$ **(D)**	0.4	0.3	0.6	0.8	0.4	0.3	0.2	0.2
$\|\mu_c\|$ **(D)**	0.6	0.7	0.7	0.7	1.1	1.1	1.0	1.0
ΔE[d] **(cm^{-1})**	2.6	40.2	19.2	10.7	45.6	39.5	73.4	61.9
ΔE_{ZPC}[e] **(cm^{-1})**	0.0	21.5	0.9	0.0	26.3	39.7	42.8	59.0
ΔE_{CP}[f] **(cm^{-1})**	10.6	66.7	41.3	60.7	64.7	71.1	78.0	38.2
Top View	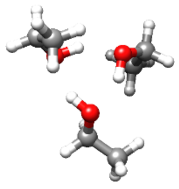		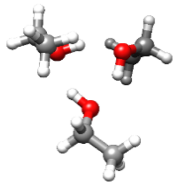		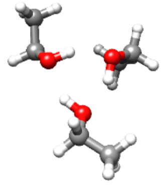		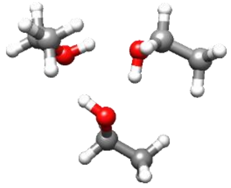	

	g−g+t(V)		*g−g+t*(VI)		*g−g+t*(VII)	
	**B3LYP**	**MP2**	**B3LYP**	**MP2**	**B3LYP**	**MP2**
A[a] **(MHz)**	1307.8	1279.5	1118.5	1150.2	1343.5	1335.9
B **(MHz)**	693.8	704.4	749.0	752.8	797.3	783.3
C **(MHz)**	477.8	481.0	476.8	487.2	606.2	590.0
**κ**[b]	-0.48	-0.44	-0.15	-0.20	-0.48	-0.48
$\|\mu_a\|$[c]**(D)**	0.3	0.3	0.1	0.2	0.1	0.2
$\|\mu_b\|$ **(D)**	0.2	0.3	0.3	0.3	0.7	0.7
$\|\mu_c\|$ **(D)**	1.1	1.0	1.1	1.2	3.3	3.2
ΔE[d] **(cm^{-1})**	142.4	118.0	152.8	104.2	261.3	293.5
ΔE_{ZPC}[e] **(cm^{-1})**	87.4	104.7	94.6	99.9	229.4	231.3
ΔE_{CP}[f] **(cm^{-1})**	152.9	87.2	160.6	68.6	290.9	355.4
Top View	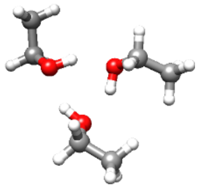		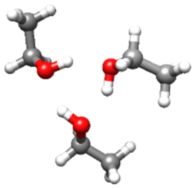		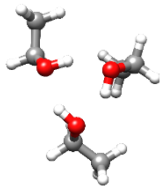	

[a] *A, B* and *C* are the rotational constants; [b] κ is Ray's asymmetry parameter; [c] $|\mu_a|$, $|\mu_b|$ and $|\mu_c|$ are the absolute values of the electric dipole moment components along the principal inertial axes; [d]ΔE are the relative energies; [e] ΔE_{ZPC} are the relative energies including zero-point corrections; [f] ΔE_{CP} are the counterpoise corrected energies; [g] Isomers marked with an asterix (*) were used as starting structures for a CREST conformational search.

Table S3. Calculated spectroscopic parameters for the *g–g–t* (*g+g+t*) isomers at the MP2/6-311++G(d,p) and B3LYP-D3BJ/6-311++G(d,p) levels of theory.

	***g–g–t*(I) *[g]**		***g–g–t*(II)**		***g–g–t*(III)**		***g–g–t*(IV)**	
	**B3LYP**	**MP2**	**B3LYP**	**MP2**	**B3LYP**	**MP2**	**B3LYP**	**MP2**
**A^{a} (MHz)**	1401.2	1405.3	1094.4	1101.8	1229.8	1207.0	1144.5	1167.0
**B (MHz)**	707.5	708.6	842.7	845.7	775.4	785.7	770.3	760.8
**C (MHz)**	522.7	524.2	542.7	550.3	535.0	534.4	513.8	515.3
**κ^{b}**	-0.58	-0.58	0.09	0.07	-0.31	-0.25	-0.19	-0.25
**$\|\mu_a\|^{c}$(D)**	0.3	0.3	0.1	0.1	0.7	0.7	0.1	0.2
**$\|\mu_b\|$ (D)**	0.5	0.5	0.4	0.5	0.1	0.2	0.7	0.6
**$\|\mu_c\|$ (D)**	1.1	1.1	1.2	1.2	0.6	0.5	0.6	0.6
**ΔE^{d} (cm^{-1})**	52.2	61.8	55.2	24.6	86.2	76.5	106.1	73.7
**ΔE_{ZPC}^{e} (cm^{-1})**	31.8	55.1	31.6	28.1	43.9	53.6	59.7	57.1
**ΔE_{CP}^{f} (cm^{-1})**	61.1	76.3	76.3	57.3	96.3	76.4	117.1	56.9
Top View	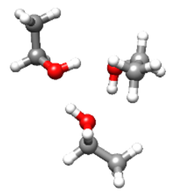		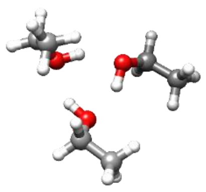		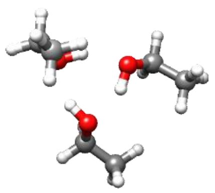		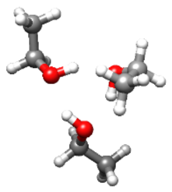	

	***g–g–t*(V)**		***g–g–t*(VI)**		***g–g–t*(VII)**		***g–g–t*(VIII)**	
	**B3LYP**	**MP2**	**B3LYP**	**MP2**	**B3LYP**	**MP2**	**B3LYP**	**MP2**
**A^{a} (MHz)**	1250.9	1321.5	1190.1	1214.7	1156.0	1148.0	1246.5	Relaxed to ***g–g–t*(III)** during optimisation
**B (MHz)**	764.3	737.4	723.8	709.8	877.9	860.4	763.9	
**C (MHz)**	540.2	527.8	473.3	476.6	643.1	620.1	524.6	
**κ^{b}**	-0.37	-0.47	-0.30	-0.37	-0.08	-0.09	-0.34	
**$\|\mu_a\|^{c}$(D)**	0.6	0.6	0.2	0.4	0.1	0.2	0.2	
**$\|\mu_b\|$ (D)**	0.0	0.2	0.3	0.2	0.4	0.5	0.1	
**$\|\mu_c\|$ (D)**	0.7	0.3	1.0	0.6	3.1	3.0	3.3	
**ΔE^{d} (cm^{-1})**	149.5	93.1	157.2	108.1	292.1	338.5	425.3	
**ΔE_{ZPC}^{e} (cm^{-1})**	106.4	72.0	96.8	99.9	247.3	263.4	318.2	
**ΔE_{CP}^{f} (cm^{-1})**	149.1	70.2	161.6	54.7	308.3	320.6	446.2	
Top View	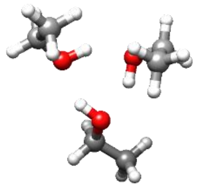		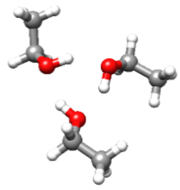		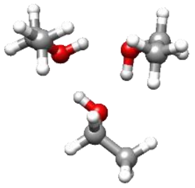		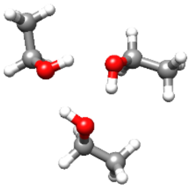	

[a] *A, B* and *C* are the rotational constants; [b] κ is Ray's asymmetry parameter; [c] $|\mu_a|$, $|\mu_b|$ and $|\mu_c|$ are the absolute values of the electric dipole moment components along the principal inertial axes; [d]ΔE are the relative energies; [e] ΔE_{ZPC} are the relative energies including zero-point corrections; [f] ΔE_{CP} are the counterpoise corrected energies; [g] Isomers marked with an asterix (*) were used as starting structures for a CREST conformational search.

Table S4. Calculated spectroscopic parameters for the *g+g+g−* (*g−g−g+*) isomers at the MP2/6-311++G(d,p) and B3LYP-D3BJ/6-311++G(d,p) levels of theory.

	***g+g+g−*(I) *[g]**		***g+g+g−*(II)**		***g+g+g−*(III)**		***g+g+g−*(IV)**	
	**B3LYP**	**MP2**	**B3LYP**	**MP2**	**B3LYP**	**MP2**	**B3LYP**	**MP2**
**A^a (MHz)**	1233.0	1176.2	997.1	1007.4	1234.1	1195.5	1059.6	1053.4
**B (MHz)**	815.8	837.3	959.1	952.2	760.5	779.0	834.0	846.4
**C (MHz)**	575.9	576.1	564.9	562.9	529.9	534.1	521.0	521.8
**κ^b**	-0.27	-0.13	0.82	0.75	-0.35	-0.26	0.16	0.22
**$\|\mu_a\|^c$(D)**	0.7	0.7	0.7	0.6	0.4	0.3	0.2	0.1
**$\|\mu_b\|$ (D)**	0.2	0.1	0.3	0.4	0.0	0.0	0.4	0.5
**$\|\mu_c\|$ (D)**	0.7	0.7	0.6	0.6	1.2	1.2	1.1	1.1
**ΔE^d (cm^{-1})**	0.0	0.0	34.1	30.0	51.0	37.1	92.4	76.0
**ΔE_{ZPC}^e (cm^{-1})**	11.4	30.5	33.8	61.2	55.7	82.7	73.3	114.3
**ΔE_{CP}^f (cm^{-1})**	0.0	10.9	25.4	0.0	51.7	24.6	81.4	9.8
Top View	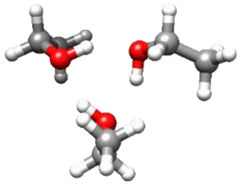		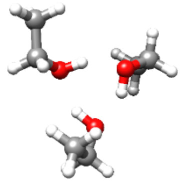		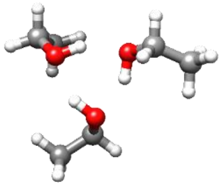		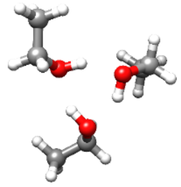	

	***g+g+g−*(V)**		***g+g+g−*(VI)**		***g+g+g−*(VII)**		***g+g+g−*(VIII)**	
	**B3LYP**	**MP2**	**B3LYP**	**MP2**	**B3LYP**	**MP2**	**B3LYP**	**MP2**
**A^a (MHz)**	1143.5	1152.8	960.4	956.6	1146.3	1146.0	1153.8	1153.4
**B (MHz)**	874.3	865.6	833.2	849.6	896.9	888.8	883.8	879.2
**C (MHz)**	588.3	580.9	470.9	476.0	649.7	632.8	607.7	599.3
**κ^b**	0.03	0.00	0.48	0.55	0.00	0.00	0.01	0.01
**$\|\mu_a\|^c$(D)**	0.8	0.7	0.3	0.2	0.2	0.2	0.3	0.4
**$\|\mu_b\|$ (D)**	0.2	0.3	0.0	0.2	0.3	0.3	0.2	0.3
**$\|\mu_c\|$ (D)**	0.4	0.4	1.1	1.1	3.2	3.0	3.4	3.3
**ΔE^d (cm^{-1})**	106.8	76.9	177.7	136.7	287.6	283.8	295.4	298.7
**ΔE_{ZPC}^e (cm^{-1})**	90.9	93.3	138.0	167.5	246.7	253.9	272.1	281.6
**ΔE_{CP}^f (cm^{-1})**	94.2	35.9	166.3	42.4	277.6	234.4	297.9	291.6
Top View	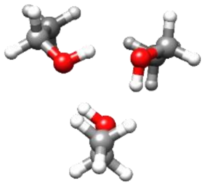		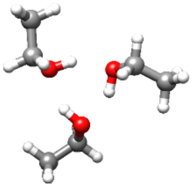		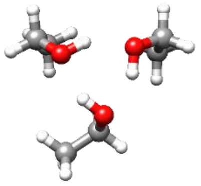		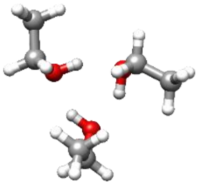	

[a] *A, B* and *C* are the rotational constants; [b] κ is Ray's asymmetry parameter; [c] $|\mu_a|$, $|\mu_b|$ and $|\mu_c|$ are the absolute values of the electric dipole moment componentss along the principal inertial axes; [d] ΔE are the relative energies; [e] ΔE_{ZPC} are the relative energies including zero-point corrections; [f] ΔE_{CP} are the counterpoise corrected energies; [g] Isomers marked with an asterix (*) were used as starting structures for a CREST conformational search.

Table S5. Calculated spectroscopic parameters for the homochiral *g+g+g+*(*g−g−g−*) and *ttt* ethanol trimer isomers at the MP2/6-311++G(d,p) and B3LYP-D3BJ/6-311++G(d,p) levels of theory.

	***g+g+g+*(I)**		***g+g+g+*(II)**		***ttt***		***g+g+g+*(III)**	
	**B3LYP**	**MP2**	**B3LYP**	**MP2**	**B3LYP**	**MP2**	**B3LYP**	**MP2**
***A*[a] (MHz)**	1021.0	1038.9	1136.1	1142.3	1013.0	1032.6	1005.5	1001.8
***B* (MHz)**	853.2	841.8	808.0	808.4	809.9	807.4	1003.8	1001.8
***C* (MHz)**	519.7	520.7	547.6	541.9	475.6	481.1	682.6	667.5
**κ[b]**	0.33	0.24	-0.11	-0.11	0.24	0.18	0.99	1.00
**$\lvert\mu_a\rvert$[c](D)**	0.4	0.4	0.5	0.4	0.4	0.3	0.0	0.0
**$\lvert\mu_b\rvert$ (D)**	0.5	0.4	0.5	0.5	0.1	0.2	0.0	0.0
**$\lvert\mu_c\rvert$ (D)**	0.6	0.5	0.8	0.7	1.1	1.2	3.0	2.8
**ΔE[d] (cm^{-1})**	116.0	77.0	159.0	109.3	171.5	148.1	428.7	378.2
**ΔE_{ZPC}[e] (cm^{-1})**	84.5	101.6	125.3	133.2	74.8	50.7	360.2	332.1
**ΔE_{CP}[f] (cm^{-1})**	100.5	20.0	138.8	15.2	214.6	206.4	409.9	298.5
Top View	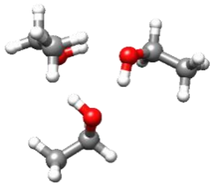		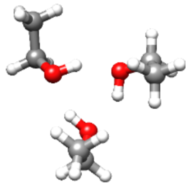		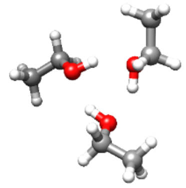		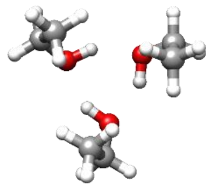	

	***g+g+g+*(IV)**	
	**B3LYP**	**MP2**
***A*[a] (MHz)**	962.1	975.1
***B* (MHz)**	960.6	974.7
***C* (MHz)**	539.4	548.1
**κ[b]**	0.99	1.00
**$\lvert\mu_a\rvert$[c](D)**	0.0	0.0
**$\lvert\mu_b\rvert$ (D)**	0.0	0.0
**$\lvert\mu_c\rvert$ (D)**	3.4	3.4
**ΔE[d] (cm^{-1})**	457.7	425.0
**ΔE_{ZPC}[e] (cm^{-1})**	377.5	381.7
**ΔE_{CP}[f] (cm^{-1})**	451.6	391.1
Top View	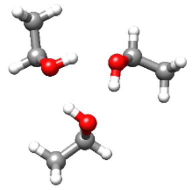	

[a] *A, B* and *C* are the rotational constants; [b] κ is the calculated Ray's asymmetry parameter; [c] $\lvert\mu_a\rvert$, $\lvert\mu_b\rvert$ and $\lvert\mu_c\rvert$ are the absolute values of the electric dipole moment components along the principal inertial axes; [d]ΔE are the relative energies; [e] ΔE_{ZPC} are the relative energies including zero-point corrections; [f] ΔE_{CP} are the counterpoise corrected energies.

S3. Assignment

Identifying the observed ethanol trimers was complex and required multiple approaches. We first compared experimental and theoretical rotational constants. Isomers 1 and 2 have very similar rotational constants but differ in the type of transitions observed. For isomer 1, *a*-, *b*- and *c*-type transitions were measured, while only *a*- and *c*-type transitions were detected for isomer 2. Looking at the theoretical rotational constants, they could be assigned to ***g+g+g−*(I)** or ***g−g+t*(I)**. Considering the differences between experimental and theoretical rotational constants and planar moments (Table S6), we assign isomer 1 to ***g−g+t*(I)** and isomer 2 to ***g+g+g−*(I)**. The very low predicted value of μ_b for ***g+g+g−*(I)** is consistent with the non-observation of *b*-type transitions for isomer 2.

Isomer 3 has lower *B* and *C* rotational constants than isomers 1 and 2, and exhibits *a*- and *c*-type spectra with the *c*-type spectrum being the most intense. Based upon the rotational constants and types of lines observed isomer 3 could correspond to ***g+g+g−*(III)**, ***g−tt*(I)**, ***g−g−t*(III)** or ***g−g−t*(V)**. However, ***g−g−t*(III)** and ***g−g−t*(V)** have similar values for μ_a and μ_c, which would have resulted in comparable intensities for *a*- and *c*-type lines. This is non consistent with observations and therefore we can disregard ***g−g−t*(III)** and ***g−g−t*(V)**. Of the other two isomers, ***g−tt*(I)** is predicted to be lower in energy by both B3LYP-D3BJ and MP2, and shows the best agreement between experimental and theoretical planar moments, and thus we would assign isomer 3 to ***g−tt*(I)**.

Table S6. Comparison of the experimental and theoretical planar moments for the potential assignments of each isomer of ethanol trimer observed in this study.

	Isomer 1	*g−g+t*(I)		*g+g+g−*(I)	
	Exp.	B3LYP	MP2	B3LYP	MP2
A[a]	1217.4761(19)	1271.9	1253.3	1233.0	1176.2
B	807.11975(49)	799.7	800.0	815.8	837.3
C	565.11681(27)	572.4	569.5	575.9	576.1
P_{aa} [b]	552.66939(99)	558.77	557.95	543.58	525.58
P_{bb}	341.62199(99)	324.15	329.46	333.97	351.67
P_{cc}	73.48192(99)	73.20	73.78	75.91	78.01
% error P_{aa}[c]		1.1	1.1	1.6	4.9
% error P_{bb}		5.1	3.6	2.2	2.9
% error P_{cc}		0.4	0.4	3.3	6.2
	**Isomer 2**	***g+g+g−*(I)**		***g−g+t*(I)**	
	**Exp.**	**B3LYP**	**MP2**	**B3LYP**	**MP2**
A[a]	1200.01318(77)	1233.0	1176.2	1271.9	1253.3
B	820.05962(57)	815.8	837.3	799.7	800.0
C	571.24062(50)	575.9	576.1	572.4	569.5
P_{aa} [b]	539.91548(54)	543.58	525.58	558.77	557.95
P_{bb}	344.78893(54)	333.97	351.67	324.15	329.46
P_{cc}	76.35568(54)	75.91	78.01	73.20	73.78
% error P_{aa}[c]		0.7	2.7	3.5	3.3
% error P_{bb}		3.1	2.0	6.0	4.4
% error P_{cc}		0.6	2.2	4.1	3.4

[a] *A, B* and *C* are the rotational constants given in MHz. [b] $P_{aa,}$ P_{bb} and P_{cc} are the planar moments in uÅ². [c] Percentage difference between the experimental and theoretical second moments calculated as $\frac{|\mathrm{exp-theo}|}{\mathrm{exp}} \times 100$.

Table S6. (cont.) Comparison of the experimental and theoretical planar moments for the potential assignments of each isomer of ethanol trimer observed in this study.

	Isomer 3	*g–tt*(I)		*g+g+g–*(III)		*g–g–t*(III)		*g–g–t*(V)	
	**Exp.**	**B3LYP**	**MP2**	**B3LYP**	**MP2**	**B3LYP**	**MP2**	**B3LYP**	**MP2**
A^a	1211.3135(11)	1265.8	1229.5	1234.1	1195.5	1229.8	1207.0	1250.9	1321.5
B	752.78429(53)	748.1	760.8	760.5	779.0	775.4	785.7	764.3	737.4
C	514.44626(39)	522.2	521.1	529.9	534.1	535.0	534.4	540.2	527.8
P_{aa} ^{b}	618.25282(64)	622.04	611.53	604.37	586.12	592.73	585.10	596.38	630.22
P_{bb}	364.12209(64)	345.75	358.30	349.35	360.10	351.91	360.59	339.16	327.30
P_{cc}	53.09368(64)	53.51	52.74	60.16	62.63	59.04	58.12	64.85	55.13
% error P_{aa}^{c}		0.6	1.1	2.2	5.2	4.1	5.4	3.5	1.9
% error P_{bb}		5.0	1.6	4.1	1.1	3.4	1.0	6.9	10.1
% error P_{cc}		0.8	0.7	13.3	18.0	11.2	9.5	22.1	3.8

	Isomer 4	*g–g–t*(II)		*g+g+g–*(IV)	
	**Exp.**	**B3LYP**	**MP2**	**B3LYP**	**MP2**
A^a	1086.27337(10)	1094.4	1101.8	1059.6	1053.4
B	833.52668(61)	842.7	845.7	834.0	846.4
C	535.45543(43)	542.7	550.3	521.0	521.8
P_{aa} ^{b}	542.45172(38)	534.58	528.64	549.52	542.93
P_{bb}	401.37865(38)	396.65	389.73	420.50	425.60
P_{cc}	63.86252(38)	65.13	68.95	56.45	54.16
% error P_{aa}^{c}		1.5	2.5	1.3	0.1
% error P_{bb}		1.2	2.9	4.8	6.0
% error P_{cc}		2.0	8.0	11.6	15.2

[a] *A, B* and *C* are the rotational constants given in MHz. [b] P_{aa}, P_{bb} and P_{cc} are the planar moments in uÅ^2. [c] Percentage difference between the experimental and theoretical second moments calculated as $\frac{|\mathrm{exp}-\mathrm{theo}|}{\mathrm{exp}} \times 100$.

Isomer 4 has an *A* rotational constant significantly different from all the other observed isomers, and shows *b*- and *c*-type lines, where *c*-type lines are stronger. Considering this, isomer 4 could correspond to ***g–g–t*(II)** or ***g+g+g–*(IV)**. Both have comparable values predicted for the dipole moment components. Since ***g–g–t*(II)** is predicted to be lower in energy both by B3LYP-D3BJ and MP2, and shows the best agreement with the calculated planar moments, particularly for B3LYP-D3BJ, we propose to assign isomer 4 to ***g–g–t*(II)**.

We tried to confirm the above assignments by running experiments with a 40:60 mixture of isotopically substituted 1-^{13}C-ethanol and parent ethanol to determine the position of the C atom in alpha with respect to the hydroxyl group for the observed isomers. It was only possible to detect the transitions corresponding to all 1-^{13}C monosubstituted isotopologues for isomer 1. From the fits of the measured transitions (see Tables S16-S18) to the same Hamiltonian used for parent species, the experimental rotational constants were determined (see Table S7). Unfortunately, the coordinates of the C_1 atom in the principal inertial axis system are very close for ***g+g+g–*(I)** and ***g–g+t*(I)** (Table S8), and it is not possible to confirm our assignment of isomer 1 based on this since we only observed one set of transitions.

Table S7. Experimental spectroscopic parameters of the observed 1-^{13}C isotopologues of isomer 1 of ethanol trimer. 1-^{13}C labelling is given in Figure S2.

Parameter	First 1-^{13}C	Second 1-^{13}C	Third 1-^{13}C
A^{a} **(MHz)**	1207.4817(27)^{b}	1199.5584(91)	1216.1885(66)
B **(MHz)**	803.23159(39)	805.75304(82)	795.64311(88)
C **(MHz)**	561.33130(18)	560.82809(44)	559.57454(35)
$\boldsymbol{\Delta_J}^{c}$ **(kHz)**	[0.420]^{d}	[0.420]	[0.420]
$\boldsymbol{\Delta_{JK}}$ **(kHz)**	[0.334]	[0.334]	[0.334]
$\boldsymbol{\Delta_K}$ **(kHz)**	[1.69]	[1.69]	[1.69]
$\boldsymbol{\delta_J}$ **(kHz)**	[0.1141]	[0.1141]	[0.1141]
$\boldsymbol{\delta_K}$ **(kHz)**	[0.435]	[0.435]	[0.435]
$\boldsymbol{\sigma}^{e}$ **(kHz)**	2.1	4.3	4.9
**N**^{f}	12	11	13

[a] *A, B* and *C* are the rotational constants. [b] Standard error in parentheses in units of the last digit. [c] Δ_J Δ_{JK} Δ_K δ_J and δ_K are the centrifugal distortion constants. [d] Parameters in brackets are fixed to the value of the parent species [e] σ is the rms deviation of the fit. [f] N is the number of fitted transitions.

Table S8. Experimental coordinates of the 1-^{13}C atoms for isomer 1 of ethanol trimer compared with equilibrium coordinates from possible structures from B3LYP-D3BJ and MP2 calculations with the 6-311++G(d,p) basis set. 1-^{13}C labelling is given in Figure S2.

	**Isomer 1**	***g−g+t*(I)**	***g−g+t*(I)**	***g+g+g−*(I)**	***g+g+g−*(I)**
	r_s	B3LYP-D3BJ	MP2	B3LYP-D3BJ	MP2
First 1-^{13}C					
$\|a\|^{a}$	1.66723(92)^{b}	1.636	1.739	1.704	1.780
$\|b\|$	1.80854(85)	1.795	1.786	1.722	1.717
$\|c\|$	0.4719(33)	0.470	0.448	0.556	0.623
Second 1-^{13}C					
$\|a\|$	0.9105(19)	0.961	0.814	0.889	0.867
$\|b\|$	2.45587(71)	2.389	2.458	2.420	2.444
$\|c\|$	0.4667(37)	0.486	0.458	0.519	0.582
Third 1-^{13}C					
$\|a\|$	2.95767(55)	2.959	2.936	2.932	2.927
$\|b\|$	0.3720(44)	0.315	0.458	0.325	0.347
$\|c\|$	0.5603(29)	0.591	0.595	0.579	0.512

^{a} Absolute values of the principal inertial axis co-ordinates in Å. ^{b} Errors include Costain's error.

Additionally, we looked at possible relaxation pathways between the low-energy isomers. Multiple structures are predicted consisting of the same ethanol monomers with different spatial arrangements. For example, the *g−tt* family has seven different isomers within 5.5 kJ mol^{-1}, and the question arises: is it possible that some of the higher energy structures relax to lower energy ones? Isomers belonging to the same family differ on having ethyl tails above or below the plane of the ring formed by the O−H···O hydrogen bonds between the hydroxyl groups. A scan through the ∠O···H-O-C dihedral angle of the ethanol monomers wihin each isomer, maintaining fixed the ∠HOCC dihedral angle of the target ethanol (to prevent interconversion between *g+*, *g−* and *t* conformations) can model the above/below-the-plane movement. It is well documented that if the barrier for interconversion is low enough, relaxation from higher to lower-energy isomers can occur by collisions at the onset of the supersonic expansion.[17,18] We performed many scans to investigate possible relaxations between isomers in the *g+g+g−*, *g−g−t*, *g−tt* and *g−g+t* families, to which our observed complexes belong. All predicted barriers at B3LYP-D3BJ/6-311++G(d,p) level are given in Table S9.

Table S9. Interconversion barrier heights calculated at the B3LYP-D3BJ/6-311++G(d,p) level of theory.

Starting structure	End Structure	Barrier (cm^{-1})
	g+g+g−	
g+g+g−(V)	*g+g+g−*(I)	93
g+g+g−(V)	*g+g+g−*(II)	70
g+g+g−(VI)	*g+g+g−*(IV)	99
g+g+g−(III)	*g+g+g−*(I)	193
g+g+g−(IV)	*g+g+g−*(II)	143
g+g+g−(VII)	*g+g+g−*(V)	254
	g−g−t	
g−g−t(VI)	*g−g−t*(I)	98
g−g−t(VI)	*g−g−t*(II)	87
g−g−t(VII)	*g−g−t*(I)	31
g−g−t(IV)	*g−g−t*(I)	129
g−g−t(VIII)	*g−g−t*(IV)	43
g−g−t(VIII)	*g−g−t*(III)	14
g−g−t(V)	*g−g−t*(III)	76
g−g−t(III)	*g−g−t*(II)	147
	g−g+t	
g−g+t(V)	*g−g+t*(V)	139
g−g+t(V)	*g−g+t*(II)	183
g−g+t(I)	*g−g+t*(I)	149
g−g+t(VI)	*g−g+t*(V)	118
g−g+t(II)	*g−g+t*(I)	182
g−g+t(VII)	*g−g+t*(I)	83
	g−tt	
g−tt(III)	*g−tt*(II)	123
g−tt(VI)	*g−tt*(V)	63
g−tt(VI)	*g−tt*(I)	86
g−tt(IV)	*g−tt*(II)	129
g−tt(III)	*g−tt*(I)	151
g−tt(V)	*g−tt*(IV)	131
g−tt(VII)	*g−tt*(II)	99

The barriers for interconversion between isomers are consistently predicted to be below 300 cm^{-1} (see

Table S9), sufficiently low for higher-energy isomers to relax to lower-energy ones. Considering these data, looking at the possible matches for isomer 3, ***g+g+g−*(III)** can relax to ***g+g+g−*(I)**. This supports our assignment of isomer 3 to ***g−tt*(I)**. Similarly, ***g+g+g−*(IV)** can relax to ***g+g+g−*(II)**, which supports our assignment of isomer 4 to ***g−g−t*(II)**.

Our investigation of possible interconversion pathways results in most higher-energy isomers being likely to relax to lower-energy isomers through low-energy barriers in our supersonic jet. In the heterochiral group *g−g+t*, the two lowest-energy structures, ***g−g+t*(II)** and ***g−g+t*(I)**, are almost isoenergetic. We looked for ***g−g+t*(II)** in our rotational spectrum but we did not observe it. After exploring the potential energy barrier for ***g−g+t*(II)** ↔ ***g−g+t*(I)** interconversion, ***g−g+t*(II)** could relax to ***g−g+t*(I)** through a low barrier of 182 cm^{-1}. The *g−g−t* and *g−tt* families also have the two lowest energy structures being almost isoenergetic. However, in these cases there is no straightforward transformation between the two minima as this would require two simultaneous motions. ***g−g−t*(I)** and ***g−tt*(II)**, the lowest and second lowest-energy isomers of the *g+g+t* and the *g+tt* families, respectively, have also been searched for in our rotational spectrum but have not been detected. We also looked for the lowest energy *g+g+g+* isomer. Although ***g+g+t*(I)** has low dipole moment components, they are comparable to μ_b of the least abundant isomer ***g−g−t*(II)**, for which *b*-type transitions were observed. Thus it is not unreasonable to assume that we could observe ***g+g+t*(I)** if it were sufficiently populated within our supersonic jet. However, no transitions were found that could match its expected spectral patterns.

The anharmonic vibrational corrections, calculated using two different approaches, also support our assignment (see Tables S10 and S11).

Table S10. Comparison of the theoretical equilibrium and ground state rotational constants (in MHz) with the experimental values for the isomers of the ethanol trimer. The equilibrium constants are at the B3LYP/6-311+G(d,p) level of theory and the vibrational corrections were calculated at the B3LYP/6-311++G(d,p) level on the BPCS-corrected equilibrium structures.

	**Isomer 1**	***g−g+t*(I)**			***g+g+g−*(I)**		
	**Exp.**	B_e	B_0	***%error***	B_e	B_0	***%error***
A	1217.4761(19)	1271.9	1205.3	1.0	1233.0	1197.5	1.6
B	807.11975(49)	799.7	793.9	1.6	815.8	804.9	0.3
C	565.11681(27)	572.4	554.8	1.8	575.9	564.6	0.1
	**Isomer 2**	***g+g+g−*(I)**			***g−g+t*(I)**		
	**Exp.**	B_e	B_0	***%error***	B_e	B_0	***%error***
A	1200.01318(77)	1233.0	1197.5	0.2	1271.9	1205.3	-0.4
B	820.05962(57)	815.8	804.9	1.8	799.7	793.9	3.2
C	571.24062(50)	575.9	564.6	1.2	572.4	554.8	2.9
	**Isomer 3**	***g−tt*(I)**			***g+g+g−*(III)**		
	**Exp.**	B_e	B_0	***%error***	B_e	B_0	***%error***
A[a]	1211.3135(11)	1265.8	1214.7	-0.3	1234.1	1202.2	0.8
B	752.78429(53)	748.1	753.2	-0.1	760.5	743.9	1.2
C	514.44626(39)	522.2	515.2	-0.1	529.9	515.0	-0.1
	**Isomer 4**	***g−g−t*(II)**			***g+g+g−*(IV)**		
	**Exp.**	B_e	B_0	***%error***	B_e	B_0	***%error***
A	1086.27337(10)	1094.4	1079.8	0.6	1059.6	1032.2	5.0
B	833.52668(61)	842.7	824.6	1.1	834.0	822.1	1.4
C	535.45543(43)	542.7	531.7	0.7	521.0	508.4	5.1

Table S11. Comparison of the theoretical equilibrium and ground state rotational constants (in MHz) with the experimental values for the isomers of the ethanol trimer. The equilibrium constants are at the rDSD/cc-pVTZ-F12 level of theory and the vibrational correction at B3LYP/6-311+G(d,p) level.

	**Isomer 1**	***g−g+t*(I)**			***g+g+g−*(I)**		
	**Exp.**	B_e	B_0	*%error*	B_e	B_0	*%error*
A	1217.4761(19)	1248.8	1145.2	5.9	1247.2	1198.2	1.6
B	807.11975(49)	814.4	809.7	-0.3	823.3	809.2	-0.3
C	565.11681(27)	576.1	548.2	3.0	583.7	565.3	0.0
	**Isomer 2**	***g+g+g−*(I)**			***g−g+t*(I)**		
	**Exp.**	B_e	B_0	*%error*	B_e	B_0	*%error*
A	1200.01318(77)	1247.2	1198.2	0.2	1248.8	1145.2	4.6
B	820.05962(57)	823.3	809.2	1.3	814.4	809.7	1.3
C	571.24062(50)	583.7	565.3	1.0	576.1	548.2	4.0
	**Isomer 3**	***g−tt*(I)**			***g+g+g−*(III)**		
	**Exp.**	B_e	B_0	*%error*	B_e	B_0	*%error*
A	1211.3135(11)	1248.1	1177.0	2.8	1245.4	1161.1	4.1
B	752.78429(53)	752.2	742.1	1.4	767.4	755.0	-0.3
C	514.44626(39)	522.1	502.8	2.3	535.1	513.1	0.3
	**Isomer 4**	***g−g−t*(II)**			***g+g+g−*(IV)**		
	**Exp.**	B_e	B_0	*%error*	B_e	B_0	*%error*
A	1086.27337(10)	1107.0	1086.3	0.0	1064.7	1063.7	2.1
B	833.52668(61)	839.7	784.5	5.9	838.7	817.7	1.9
C	535.45543(43)	543.2	516.6	3.5	523.7	516.4	3.6

S4. Natural Bonding Orbital Data

Table S12. Intermolecular stabilising energy contributions (≥0.42 kJ mol^{-1}) for isomers 1 – 4 of ethanol trimer from Natural Bond Orbital (NBO) analysis at the B3LYP-D3BJ/6-311++G(d,p) level of theory.

**Isomer 1 – *g–g+t*(I)**			**Isomer 2 – *g+g+g–*(I)**		
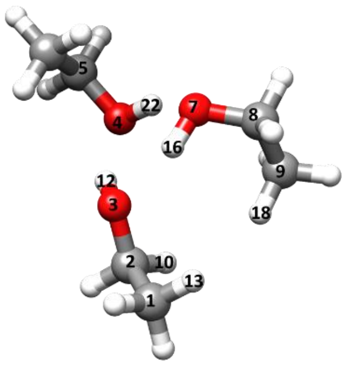			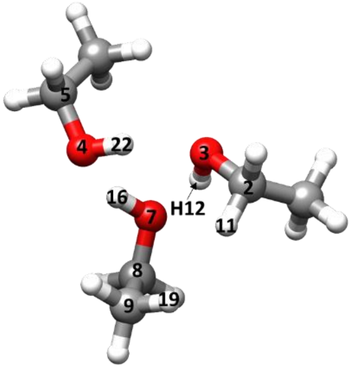		
**From unit 1 to unit 2**			**From unit 1 to unit 2**		
**Donor**	**Acceptor**	**kJ mol^{-1}**	**Donor**	**Acceptor**	**kJ mol^{-1}**
BD(1)O3-H12	BD*(1)O4-C5	0.71	BD(1)O4-H22	BD*(1)C2-O3	0.59
**From unit 1 to unit 3**			**From unit 1 to unit 3**		
**Donor**	**Acceptor**	**kJ mol^{-1}**	**Donor**	**Acceptor**	**kJ/mol**
LP(2)O3	BD*(1)O7-H16	40.38	LP(2)O4	BD*(1)O7-H16	45.44
LP(1)O3	BD*(1)O7-H16	6.23	LP(1)O4	BD*(1)O7-H16	6.11
BD(1)O3-H12	BD*(1)O7-H16	0.92	BD(1)O4-H22	BD*(1)O7-H16	1.26
LP(2)O3	RY*(5)H16	0.50	LP(2)O4	RY*(5)H16	0.50
LP(2)O3	RY*(3)O7	0.46	LP(2)O4	BD*(1)O7-C8	0.50
LP(2)O3	RY*(1)H16	0.46	LP(2)O4	RY*(4)H16	0.42
**From unit 2 to unit 1**			**From unit 2 to unit 1**		
**Donor**	**Acceptor**	**kJ mol^{-1}**	**Donor**	**Acceptor**	**kJ mol^{-1}**
LP(2)O4	BD*(1)O3-H12	44.60	LP(2)O3	BD*(1)O4-H22	43.47
LP(1)O4	BD*(1)O3-H12	5.94	LP(1)O3	BD*(1)O4-H22	5.02
BD(1)O4-H22	BD*(1)O3-H12	1.05	BD(1)O3-H12	BD*(1)O4-H22	1.05
LP(2)O4	RY*(5)H12	0.63	LP(2)O3	RY*(2)O4	0.71
LP(2)O4	BD*(1)C2-O3	0.46	LP(2)O3	RY*(4)H22	0.50
			LP(2)O3	RY*(5)H22	0.50
**From unit 2 to unit 3**			**From unit 2 to unit 3**		
**Donor**	**Acceptor**	**kJ mol^{-1}**	**Donor**	**Acceptor**	**kJ mol^{-1}**
BD(1)O4-H22	BD*(1)O7-C8	0.84	BD(1)O3-H12	BD*(1)O7-C8	0.71
LP(1)O4	BD*(1)O7-C8	0.42	BD(1)C2-H11	BD*(1)C9-H19	0.42
**From unit 3 to unit 1**			**From unit 3 to unit 1**		
**Donor**	**Acceptor**	**kJ mol^{-1}**	**Donor**	**Acceptor**	**kJ mol^{-1}**
BD(1)O7-H16	RY*(5)O3	0.71	BD(1)O7-H16	BD*(1)O4-C5	0.71
BD(1)O7-H16	BD*(1)C2-O3	0.59			
**From unit 3 to unit 2**			**From unit 3 to unit 2**		
**Donor**	**Acceptor**	**kJ mol^{-1}**	**Donor**	**Acceptor**	**kJ mol^{-1}**
LP(2)O7	BD*(1)O4-H22	39.96	LP(2)O7	BD*(1)O3-H12	39.04
LP(1)O7	BD*(1)O4-H22	7.20	LP(1)O7	BD*(1)O3-H12	7.11
BD(1)O7-H16	BD*(1)O4-H22	1.05	BD(1)O7-H16	BD*(1)O3-H12	1.13
LP(2)O7	RY*(2)O4	0.79	BD(1)C9-H19	BD*(1)C2-H11	0.63
LP(2)O7	RY*(4)H22	0.50	LP(2)O7	RY*(4)H12	0.54
LP(2)O7	RY*(5)H22	0.42	LP(2)O7	RY*(1)H12	0.50
LP(2)O7	BD*(1)O4-C5	0.42			

Isomer 3 – *g–tt*(I)			Isomer 4 – *g–g–t*(II)		
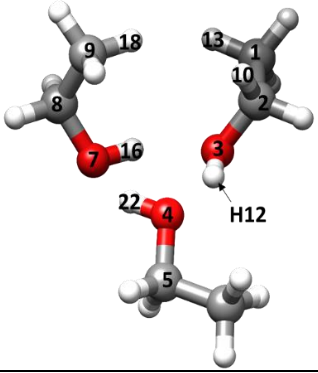			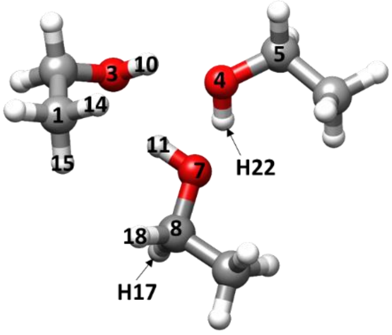		
**From unit 1 to unit 2**			**From unit 1 to unit 2**		
**Donor**	**Acceptor**	**kJ mol^{-1}**	**Donor**	**Acceptor**	**kJ mol^{-1}**
BD(1)O3-H12	RY*(5)O4	0.54	BD(1)O3-H10	BD*(1)O4-C5	0.71
BD(1)O3-H12	BD*(1)O4-C5	0.75			
**From unit 1 to unit 3**			**From unit 1 to unit 3**		
**Donor**	**Acceptor**	**kJ mol^{-1}**	**Donor**	**Acceptor**	**kJ mol^{-1}**
LP(2)O3	BD*(1)O7-H16	39.75	LP(2)O3	BD*(1)O7-H11	36.23
LP(1)O3	BD*(1)O7-H16	6.15	LP(1)O3	BD*(1)O7-H11	6.82
BD(1)O3-H12	BD*(1)O7-H16	0.96	BD(1)C1-H15	BD*(1)C8-H18	0.75
LP(2)O3	RY*(5)H16	0.50	BD(1)O3-H10	BD*(1)O7-H11	0.75
LP(2)O3	RY*(1)H16	0.46	LP(2)O3	RY*(5)H11	0.54
			LP(2)O3	RY*(1)H11	0.46
**From unit 2 to unit 1**			**From unit 2 to unit 1**		
**Donor**	**Acceptor**	**kJ mol^{-1}**	**Donor**	**Acceptor**	**kJ mol^{-1}**
LP(2)O4	BD*(1)O3-H12	45.15	LP(2)O4	BD*(1)O3-H10	43.51
LP(1)O4	BD*(1)O3-H12	6.44	LP(1)O4	BD*(1)O3-H10	5.23
BD(1)O4-H22	BD*(1)O3-H12	0.96	BD(1)O4-H22	BD*(1)O3-H10	1.21
LP(2)O4	RY*(5)H12	0.59	LP(2)O4	RY*(4)H10	0.59
LP(2)O4	RY*(3)O3	0.54	LP(2)O4	RY*(3)O3	0.50
LP(2)O4	BD*(1)C2-O3	0.46	LP(2)O4	RY*(5)H10	0.42
			LP(2)O4	BD*(1)C2-O3	0.42
**From unit 2 to unit 3**			**From unit 2 to unit 3**		
**Donor**	**Acceptor**	**kJ mol^{-1}**	**Donor**	**Acceptor**	**kJ mol^{-1}**
BD(1)O4-H22	BD*(1)O7-C8	0.84	BD(1)O4-H22	BD*(1)O7-C8	0.67
			BD(1)O4-H22	RY*(5)O7	0.63
**From unit 3 to unit 1**			**From unit 3 to unit 1**		
**Donor**	**Acceptor**	**kJ mol^{-1}**	**Donor**	**Acceptor**	**kJ mol^{-1}**
BD(1)O7-H16	RY*(5)O3	0.67	BD(1)O7-H11	BD*(1)C2-O3	0.75
BD(1)O7-H16	BD*(1)C2-O3	0.59	BD(1)C8-H18	BD*(1)C1-H15	0.59
			BD(1)O7-H11	RY*(4)O3	0.46
**From unit 3 to unit 2**			**From unit 3 to unit 2**		
**Donor**	**Acceptor**	**kJ mol^{-1}**	**Donor**	**Acceptor**	**kJ mol^{-1}**
LP(2)O7	BD*(1)O4-H22	41.00	LP(2)O7	BD*(1)O4-H22	48.37
LP(1)O7	BD*(1)O4-H22	6.99	LP(1)O7	BD*(1)O4-H22	6.69
BD(1)O7-H16	BD*(1)O4-H22	0.92	BD(1)O7-H11	BD*(1)O4-H22	1.21
LP(2)O7	RY*(5)H22	0.59	LP(2)O7	RY*(5)H22	0.59
LP(2)O7	BD*(1)O4-C5	0.50	LP(2)O7	BD*(1)O4-C5	0.46

Table S13. Intermolecular stabilising energy contributions for the H···H contacts of isomers 1 – 4 of ethanol trimer from Natural Bond Orbital (NBO) analysis at the B3LYP-D3BJ/6-311++G(d,p) level of theory.

	Donor	Acceptor	kJ·mol^{-1}	Total
g−*g*+*t*(I)	BD(1)C1-H13	BD*(1)C9-H18	0.25	1.01
	BD(1)C2-H10	BD*(1)C9-H18	0.38	
	BD(1)C9-H18	BD*(1)C2-H10	0.38	
g+*g*+*g*−(I)	BD(1)C2-H11	BD*(1)C9-H19	0.42	1.05
	BD(1)C9-H19	BD*(1)C2-H11	0.63	
g−*tt*(I)	BD(1)C1-H13	BD*(1)C9-H18	0.25	0.96
	BD(1)C2-H10	BD*(1)C9-H18	0.33	
	BD(1)C9-H18	BD*(1)C2-H10	0.38	
g−*g*−*t*(II)	BD(1)C8-H18	RY*(1)H15	0.25	1.59
	BD(1)C8-H18	BD*(1)C1-H15	0.59	
	BD(1)C1-H15	BD*(1)C8-H18	0.75	

S5. QTAIM Analysis

Figure S1. Results form the QTAIM analysis of the observed ethanol trimer isomers. Bond paths are shown in orange. Critical Points (CPs) on bond paths are bond critical points (BCPs), shown with yellow spheres and the corresponding electron densities (in a.u.) of BCPs are given. CPs not on bond paths are ring critical points (RCPs), shown with green spheres.

Isomer 1 - *g−g+t*(I)	Isomer 2 - *g+g+g−*(I)
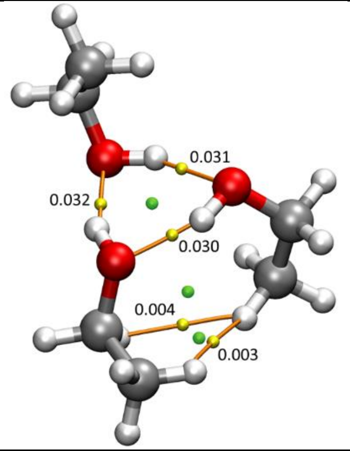	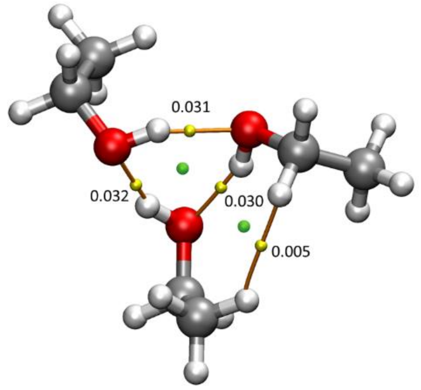
**Isomer 3 - *g−tt*(I)**	**Isomer 4 - *g−g−t*(II)**
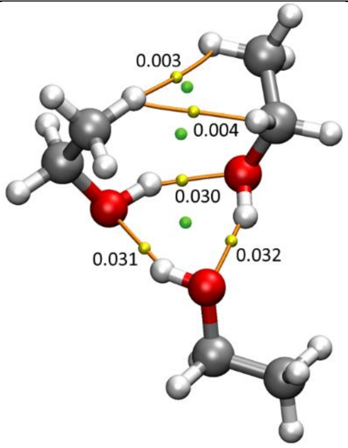	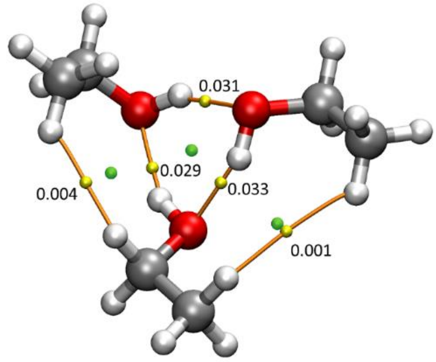

Table S14. Calculated O···O distances at B3LYP-D3BJ/6-311++G(d,p) and MP2/6-311++G(d,p) (given in brackets) levels of theory. Oxygen nomenclature is given in Figure S2.

	$O_1 \cdots O_2$	$O_2 \cdots O_3$	$O_3 \cdots O_1$	Average
***g−g+t*(I)**	2.77 (2.79)	2.78 (2.80)	2.76 (2.77)	2.77 (2.79)
***g+g+g−*(I)**	2.77 (2.79)	2.78 (2.80)	2.76 (2.78)	2.77 (2.79)
***g−tt*(I)**	2.78 (2.81)	2.75 (2.76)	2.75 (2.77)	2.76 (2.78)
***g−g−t*(II)**	2.77 (2.79)	2.75 (2.76)	2.77 (2.80)	2.76 (2.78)

Table S15. Calculated O···O distances of several alcohol trimers and the water trimer at B3LYP-D3BJ/6-311++G(d,p) and MP2/6-311++G(d,p) levels of theory.

	B3LYP-D3BJ	MP2
Water[43]	2.77	2.80
Methanol[19]	2.75	2.77
Ethanol	2.77	2.79
Phenol[20]	2.80	2.84
2,2,2-TFE-I[21]	2.75	2.78
2,2,2-TFE-II[21]	2.74	2.78

Figure S2. Oxygen labelling for the calculated O···O distances and labelling for the 1-^{13}C carbon isotopologues.

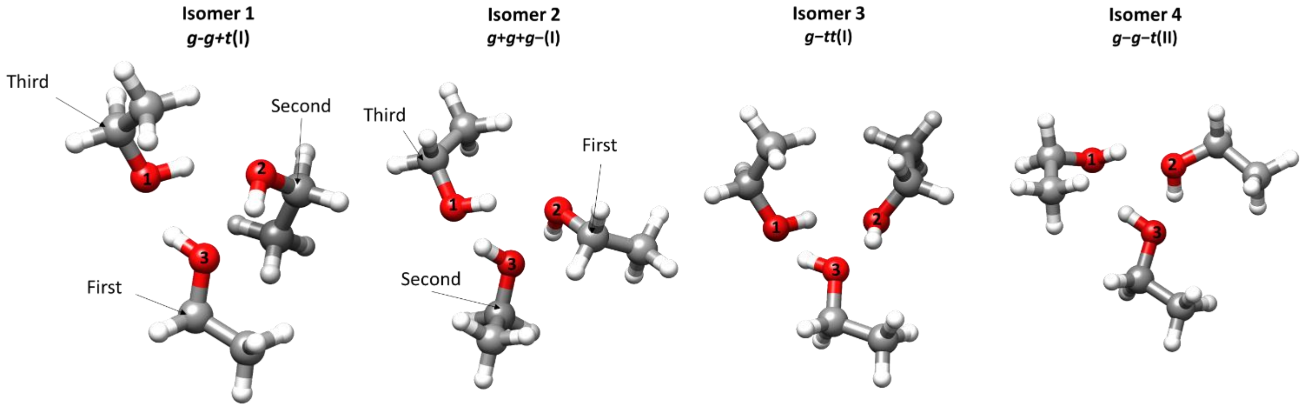

S6. Liquid Phase Results

Table S16. Calculated low-energy liquid phase structures up to 300 cm^{-1} from B3LYP-D3BJ/6-311++G(d,p) level of theory with the polarizable continuum model SMD using ethanol as a solvent. Structures named CONFX are from the CREST-CENSO calculation described in section S2, where X corresponds to the number of the output.

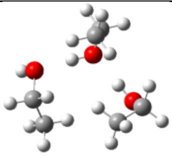<br>CONF68<br>*g+g+g+*<br>0.0	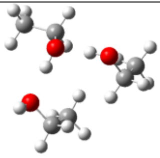<br>CONF116<br>*g+g+t*<br>67.6	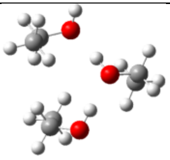<br>CONF95<br>*g-g-g+*<br>69.9
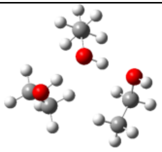<br>CONF103<br>*g+g+g+*<br>81.0	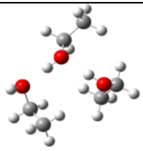<br>CONF128<br>*g-g-t*<br>86.8	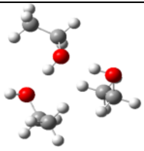<br>CONF111<br>*g-g-g+*<br>93.5
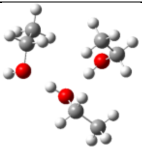<br>CONF127<br>*g-g-t*<br>96.1	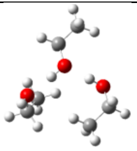<br>CONF138<br>*g-g-g+*<br>96.2	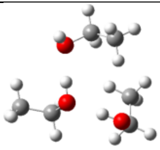<br>CONF69<br>*g-g+g+*<br>96.7
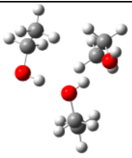<br>CONF94<br>*g-g+t*<br>111.2	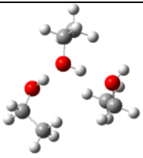<br>CONF30<br>*g-g-g+*<br>116.3	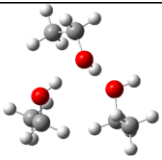<br>CONF96<br>*g-g+g+*<br>124.1
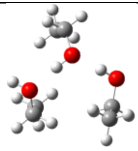<br>CONF43<br>*g-g-g+*<br>133.6	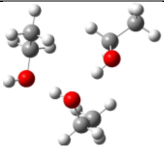<br>CONF139<br>*g+g+t*<br>134.9	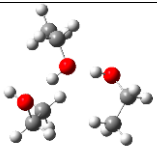<br>CONF29<br>*g-g+g+*<br>139.7
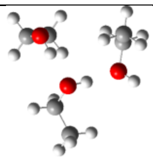<br>CONF104<br>*g-g+g+*<br>140.3	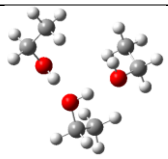<br>CONF27<br>*g-g-g+*<br>144.8	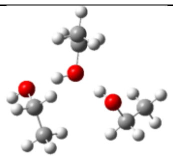<br>CONF46<br>*g+g+g+*<br>149.8
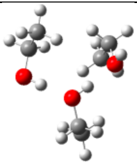<br>CONF94<br>*g-g+t*<br>111.2	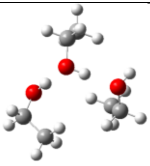<br>CONF30<br>*g-g-g+*<br>116.3	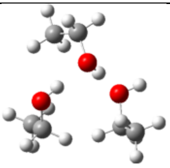<br>CONF96<br>*g-g+g+*<br>124.1

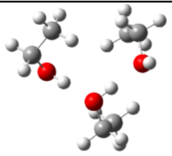<br>CONF84<br>*g-g+t*<br>150.9	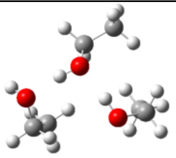<br>CONF144<br>*g-g+t*<br>155.6	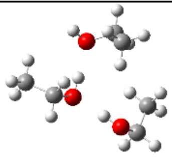<br>CONF52<br>*g+g+g+*<br>157.0
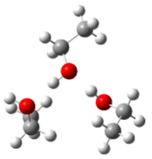<br>CONF120<br>*g+g+t*<br>159.6	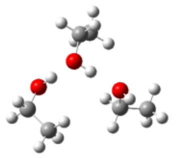<br>CONF64<br>*g-g-g+*<br>162.3	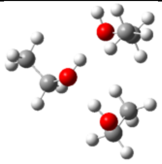<br>CONF102<br>*g-g+g+*<br>164.1
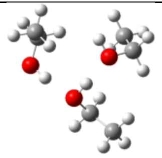<br>CONF60<br>*g-g-g+*<br>165.6	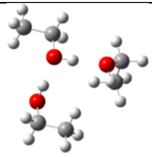<br>CONF89<br>*g-g+t*<br>167.4	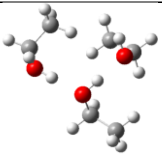<br>CONF50<br>*g-g+g+*<br>169.5
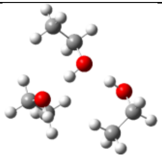<br>CONF51<br>*g-g+g+*<br>171.5	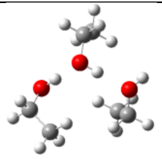<br>CONF3<br>*g-g-g+*<br>172.7	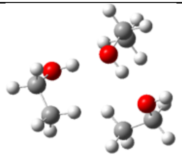<br>CONF108<br>*g-g+t*<br>174.6
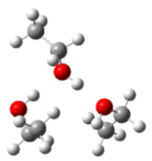<br>CONF131<br>*g+g+t*<br>181.8	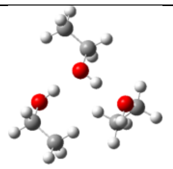<br>CONF88<br>*g-g+t*<br>182.4	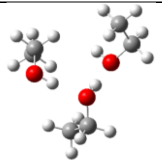<br>CONF97<br>*g-g+g+*<br>182.6
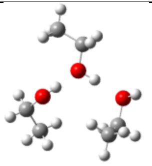<br>CONF87<br>*g+g+t*<br>183.0	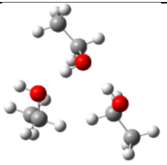<br>CONF148<br>*g+g+t*<br>183.3	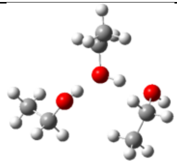<br>CONF49<br>*g-g-g-*<br>184.2
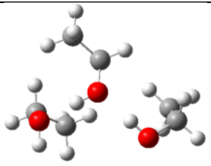<br>CONF62<br>*g-g-g+*<br>184.9	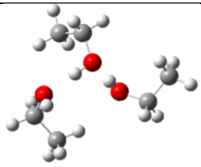<br>CONF33<br>*g-g-g-*<br>187.5	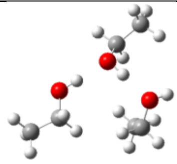<br>CONF115<br>*g-g-t*<br>190.5

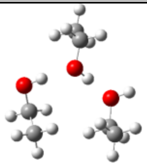<br>CONF90<br>*g+g+g+*<br>194.7	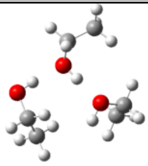<br>CONF105<br>*g-g+g+*<br>195.1	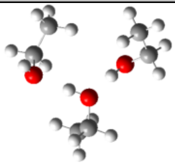<br>CONF40<br>*g-g-g+*<br>195.9
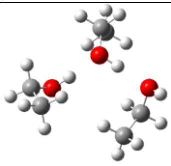<br>CONF38<br>*g-g+g+*<br>196.3	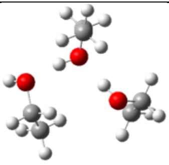<br>CONF41<br>*g-g+g+*<br>196.5	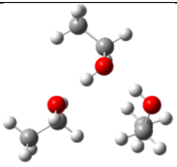<br>CONF126<br>*g+g+g+*<br>196.6
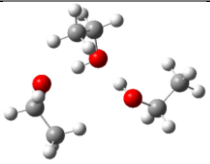<br>CONF32<br>*g+g+g+*<br>200.9	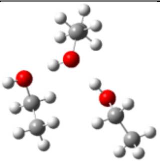<br>CONF110<br>*g-g-t*<br>203.0	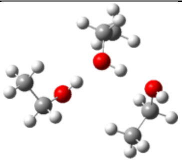<br>CONF34<br>*g+g+g+*<br>203.7
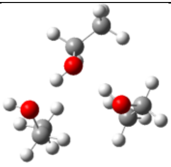<br>CONF141<br>*g-g+t*<br>206.6	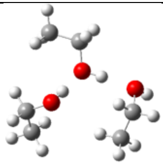<br>CONF112<br>*g-g+t*<br>207.3	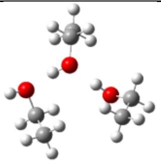<br>CONF39<br>*g-g-g+*<br>209.9
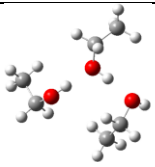<br>CONF57<br>*g-g-g+*<br>215.3	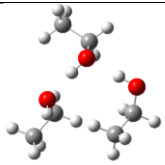<br>CONF101<br>*g-g-g+*<br>217.5	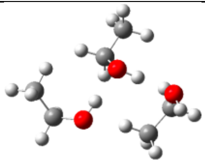<br>CONF59<br>*g-g-g+*<br>219.7
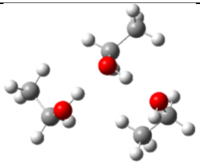<br>CONF26<br>*g-g-g+*<br>220.4	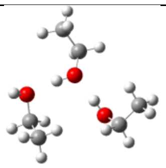<br>CONF79<br>*g-g-g+*<br>221.6	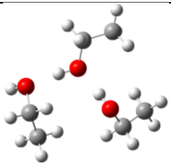<br>CONF98<br>*g+g+t*<br>224.4
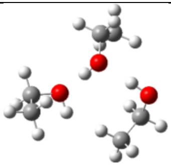<br>CONF82<br>*g-g-g+*<br>224.8	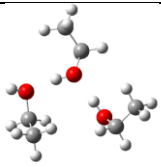<br>CONF80<br>*g-g-g+*<br>225.5	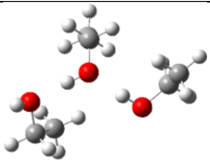<br>CONF100<br>*g-g+t*<br>225.8

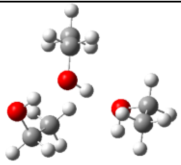<br>CONF99<br>*g-g-g+*<br>229.2	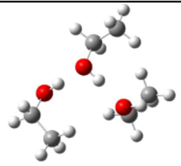<br>CONF85<br>*g-g-g-*<br>231.2	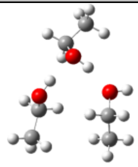<br>CONF147<br>*g-g-t*<br>231.5
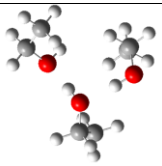<br>CONF92<br>*g-g+g+*<br>233.1	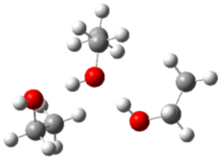<br>CONF93<br>*g-g-t*<br>234.6	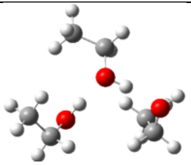<br>CONF123<br>*g-g+t*<br>236.1
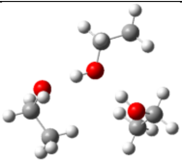<br>CONF37<br>*g-g-t*<br>239.2	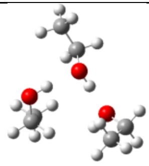<br>CONF130<br>*g-g-t*<br>239.9	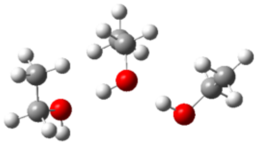<br>CONF53<br>*g-g+t*<br>247.9
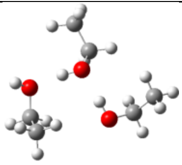<br>CONF81<br>*g-g+g+*<br>248.3	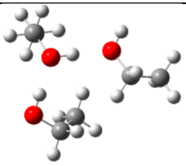<br>CONF114<br>*g-g+g+*<br>252.4	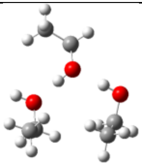<br>CONF119<br>*g-g+g+*<br>252.6
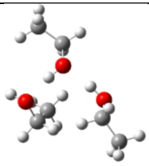<br>CONF143<br>*g+g+t*<br>252.9	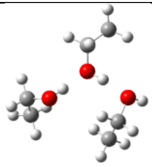<br>CONF76<br>*g-g+g+*<br>256.7	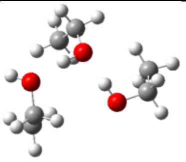<br>CONF45<br>*g-g-g+*<br>265.1
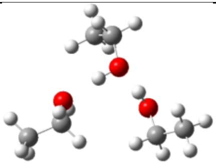<br>CONF70<br>*g-g-g+*<br>270.9	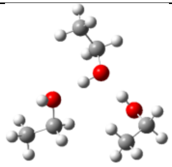<br>CONF86<br>*g+g+g+*<br>272.8	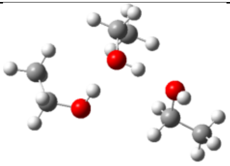<br>CONF75<br>*g-g+g+*<br>272.9
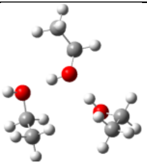<br>CONF77<br>*g-g+g+*<br>275.1	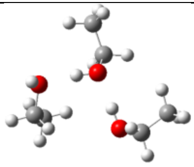<br>CONF117<br>*g-g+t*<br>277.0	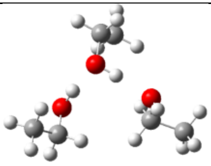<br>CONF72<br>*g-g+g+*<br>278.6

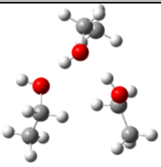<br>CONF142<br>*g-g+t*<br>280.4	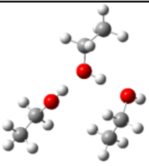<br>CONF125<br>*g-g+t*<br>281.3	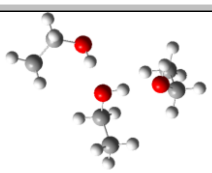<br>CONF56<br>*g-g+g+*<br>284.5
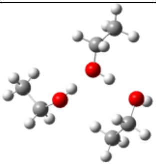<br>CONF78<br>*g-g-g+*<br>284.6	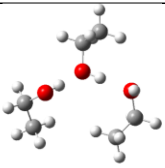<br>CONF15<br>*g+g+g+*<br>289.5	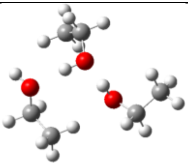<br>CONF113<br>*g-g-t*<br>295.0
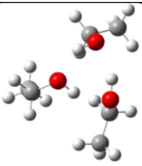<br>*g−tt*(VII)<br>*g-tt*<br>295.6	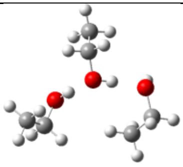<br>CONF2<br>*g-g+g+*<br>296.7	

S7. Measured frequencies

Table S17. Measured frequencies and residuals (in MHz) of the rotational transitions of the parent species of *g–g+t*(I).

J'	K'_{-1}	K'_{+1}	*J''*	K''_{-1}	K''_{+1}	ν_{obs}	ν_{obs}-ν_{calc}
1	1	0	0	0	0	2024.5857	-0.0048
3	2	2	3	0	3	2479.6373	-0.0023
2	1	2	1	1	1	2502.4616	0.0007
4	1	4	3	2	2	2520.8828	0.0135
2	0	2	1	0	1	2664.7846	-0.0009
4	2	3	4	1	4	2904.3084	-0.0010
2	1	2	1	0	1	2912.8180	0.0019
4	2	3	4	0	4	2949.2247	0.0007
2	1	1	1	1	0	2986.4553	-0.0006
5	1	5	4	2	3	3131.7397	-0.0069
5	1	4	5	1	5	3235.0581	0.0037
5	1	4	5	0	5	3250.6098	0.0039
4	0	4	3	1	2	3405.2376	-0.0035
5	2	4	5	1	5	3553.8732	0.0005
5	2	4	5	0	5	3569.4261	0.0019
3	0	3	2	1	2	3594.0487	-0.0077
2	1	1	1	0	1	3638.8097	-0.0018
3	1	3	2	1	2	3709.9336	0.0010
3	0	3	2	0	2	3842.0898	0.0028
3	1	3	2	0	2	3957.9664	0.0032
3	2	2	2	2	1	4116.6561	-0.0002
6	1	5	6	1	6	4135.1394	0.0033
6	1	5	6	0	6	4140.1722	0.0115
2	2	1	1	1	0	4217.4958	-0.0044
6	2	5	6	1	6	4277.8563	-0.0068
6	2	5	6	0	6	4282.9052	0.0175
2	2	0	1	1	0	4297.1724	-0.0020
3	2	1	2	2	0	4391.2310	-0.0027
3	1	2	2	1	1	4418.4132	-0.0011
2	2	1	1	1	1	4459.4992	-0.0012
4	1	3	3	2	1	4472.6053	0.0039
2	2	0	1	1	1	4539.1735	-0.0012
4	1	3	3	2	2	4826.8561	0.0031
4	0	4	3	1	3	4839.7193	0.0013
4	1	4	3	1	3	4884.6333	0.0006
2	2	0	1	0	1	4949.5301	0.0002
4	0	4	3	0	3	4955.5976	0.0033
4	1	4	3	0	3	5000.5102	0.0013
3	2	2	2	1	1	5347.7095	0.0088
3	1	2	2	0	2	5392.4363	-0.0040
4	2	3	3	2	2	5425.1785	-0.0001
5	1	4	4	2	2	5482.4701	0.0072
4	3	2	3	3	1	5609.0443	-0.0008
3	2	1	2	1	1	5701.9512	-0.0010
4	3	1	3	3	0	5705.4819	-0.0028
4	1	3	3	1	2	5756.1379	-0.0016
8	1	7	8	1	8	5809.9630	-0.0138
4	2	2	3	2	1	5955.2639	-0.0012
5	0	5	4	1	4	6020.5003	-0.0041
5	1	5	4	1	4	6036.0584	0.0025
5	0	5	4	0	4	6065.4224	0.0033

3	2	2	2	1	2	6073.6956	-0.0004
5	1	5	4	0	4	6080.9673	-0.0033
4	2	3	3	1	2	6354.4692	0.0042
5	1	4	4	2	3	6366.7961	-0.0048
3	2	1	2	1	2	6427.9401	-0.0075
3	2	1	2	0	2	6675.9722	-0.0060
5	2	4	4	2	3	6685.6252	0.0060
5	1	4	4	1	3	6965.1320	0.0055
5	3	3	4	3	2	7002.6020	-0.0036
5	4	2	4	4	1	7036.2613	0.0013
5	4	1	4	4	0	7060.2231	-0.0012
6	0	6	5	1	5	7169.7601	-0.0083
6	1	6	5	1	5	7174.7940	0.0010
6	0	6	5	0	5	7185.3188	-0.0011
6	1	6	5	0	5	7190.3402	-0.0043
4	2	2	3	1	2	7238.8017	-0.0013
5	3	2	4	3	1	7282.9118	-0.0014
5	2	4	4	1	3	7283.9512	0.0064
4	1	3	3	0	3	7306.4903	-0.0023
5	2	3	4	2	2	7457.2069	-0.0020
6	1	5	5	2	4	7756.0534	-0.0031
4	2	3	3	1	3	7788.9454	0.0034
6	2	5	5	2	4	7898.7924	0.0089

Table S18. Measured frequencies and residuals (in MHz) of the rotational transitions of first 1-^{13}C isotopologue of *g−g+t*(I). 1-^{13}C labelling is given in Figure S2.

J'	K'_{-1}	K'_{+1}	*J''*	K''_{-1}	K''_{+1}	ν_{obs}	ν_{obs}-ν_{calc}
2	1	2	1	1	1	2487.2177	0.0015
2	0	2	1	0	1	2648.6350	-0.0011
2	1	1	1	1	0	2971.0065	0.0006
3	1	3	2	1	2	3686.6681	-0.0023
3	0	3	2	0	2	3817.0299	-0.0003
3	1	2	2	1	1	4394.4552	-0.0029
4	1	4	3	1	3	4853.2541	0.0012
4	0	4	3	0	3	4922.5303	0.0002
4	1	3	3	1	2	5722.4307	0.0045
5	1	5	4	1	4	5996.6653	-0.0020
5	0	5	4	0	4	6025.0428	0.0022
5	1	4	4	1	3	6920.8159	-0.0021

Table S19. Measured frequencies and residuals (in MHz) of the rotational transitions of second 1-^{13}C isotopologue of *g*−*g*+*t*(I). 1-^{13}C labelling is given in Figure S2.

J'	*K'*$_{-1}$	*K'*$_{+1}$	*J''*	*K''*$_{-1}$	*K''*$_{+1}$	ν_{obs}	ν_{obs}-ν_{calc}
2	1	2	1	1	1	2488.2237	-0.0042
2	0	2	1	0	1	2649.3972	-0.0040
2	1	1	1	1	0	2978.0681	0.0010
3	1	3	2	1	2	3686.5087	-0.0093
3	0	3	2	0	2	3813.8725	-0.0020
3	1	2	2	1	1	4402.0667	0.0025
4	1	4	3	1	3	4851.2435	0.0078
4	0	4	3	0	3	4917.0702	0.0009
4	1	3	3	1	2	5725.8741	-0.0005
5	1	5	4	1	4	5992.7805	0.0024
5	0	5	4	0	4	6019.0134	-0.0008

Table S20. Measured frequencies and residuals (in MHz) of the rotational transitions of third 1-^{13}C isotopologue of *g*−*g*+*t*(I). 1-^{13}C labelling is given in Figure S2.

J'	*K'*$_{-1}$	*K'*$_{+1}$	*J''*	*K''*$_{-1}$	*K''*$_{+1}$	ν_{obs}	ν_{obs}-ν_{calc}
2	1	2	1	1	1	2474.3618	0.0028
2	0	2	1	0	1	2635.4265	-0.0048
2	1	1	1	1	0	2946.4803	-0.0033
3	1	3	2	1	2	3670.2055	-0.0023
3	0	3	2	0	2	3804.9096	0.0015
3	1	2	2	1	1	4362.5607	-0.0003
4	1	4	3	1	3	4834.4916	0.0046
4	0	4	3	0	3	4909.3660	-0.0012
4	1	3	3	1	2	5690.9126	0.0025
5	1	5	4	1	4	5975.7637	0.0037
5	0	5	4	0	4	6007.8278	-0.0065
5	1	4	4	1	3	6896.8480	0.0008
6	1	6	5	1	5	7104.0732	-0.0160

Table S21. Measured frequencies and residuals (in MHz) of the rotational transitions of the parent species of *g*+*g*+*g*−(I).

J'	K'_{-1}	K'_{+1}	J''	K''_{-1}	K''_{+1}	ν_{obs}	ν_{obs}-ν_{calc}
2	1	2	1	1	1	2533.7738	0.0019
2	0	2	1	0	1	2694.3871	0.0037
2	1	1	1	1	0	3031.3996	-0.0028
2	1	1	1	0	1	3660.1870	0.0131
3	1	3	2	1	2	3752.5690	-0.0015
3	0	3	2	0	2	3875.7476	0.0040
3	2	2	2	2	1	4173.8560	0.0030
2	2	0	1	1	0	4259.4664	-0.0011
2	2	1	1	1	1	4420.0828	0.0021
3	2	1	2	2	0	4471.9615	-0.0070
3	1	2	2	1	1	4477.9295	-0.0003
4	1	4	3	1	3	4936.9013	-0.0072
4	0	4	3	0	3	4998.2375	0.0027
3	1	2	2	0	2	5443.7188	-0.0017
4	2	3	3	2	2	5494.3238	0.0019
4	3	2	3	3	1	5695.6694	0.0015
3	2	1	2	1	1	5700.0308	-0.0028
4	3	1	3	3	0	5809.9630	-0.0098
4	1	3	3	1	2	5817.8260	0.0066
4	2	2	3	2	1	6057.0501	-0.0056
3	2	2	2	1	2	6060.1595	-0.0024
5	1	5	4	1	4	6098.1300	0.0023
5	0	5	4	0	4	6121.6885	0.0041
5	2	4	4	2	3	6762.4700	-0.0064
5	1	4	4	1	3	7019.5364	-0.0031
5	3	3	4	3	2	7105.1228	0.0045
5	4	2	4	4	1	7150.0836	-0.0061
5	4	1	4	4	0	7181.1517	0.0092
6	1	6	5	1	5	7247.6008	0.0016
6	0	6	5	0	5	7255.4667	-0.0044
4	2	2	3	1	2	7279.1540	-0.0055
4	1	3	3	0	3	7385.8037	0.0075
5	3	2	4	3	1	7426.9602	0.0065
5	2	3	4	2	2	7566.0426	-0.0054
4	2	3	3	1	3	7801.9128	-0.0005
6	2	5	5	2	4	7981.1034	0.0025

Table S22. Measured frequencies and residuals (in MHz) of the rotational transitions of the parent species of *g–tt*(I).

J'	K'_{-1}	K'_{+1}	*J''*	K''_{-1}	K''_{+1}	ν_{obs}	$\nu_{obs}-\nu_{calc}$
3	1	3	2	1	2	3404.5364	-0.0004
2	1	1	1	0	1	3469.6359	0.0004
3	0	3	2	0	2	3549.7389	-0.0004
3	2	1	2	2	0	4053.5303	-0.0006
3	1	2	2	1	1	4105.3492	0.0009
2	2	0	1	1	0	4219.8597	-0.0003
2	2	1	1	1	1	4386.6648	-0.0002
4	1	4	3	1	3	4481.7811	-0.0011
4	0	4	3	0	3	4566.8695	-0.0036
4	2	3	3	2	2	5011.9138	0.0021
3	1	2	2	0	2	5112.0665	0.0018
4	3	2	3	3	1	5179.1462	0.0055
4	3	1	3	3	0	5258.4786	-0.0045
4	1	3	3	1	2	5355.7087	-0.0019
3	2	1	2	1	1	5500.6179	0.0009
4	2	2	3	2	1	5511.5406	-0.0012
5	1	5	4	1	4	5535.4151	0.0002
5	0	5	4	0	4	5573.8637	0.0099
3	2	2	2	1	2	5892.1768	0.0000
5	2	4	4	2	3	6178.2532	0.0006
5	3	3	4	3	2	6470.8885	0.0031
5	1	4	4	1	3	6488.0458	-0.0035
5	4	2	4	4	1	6493.4529	-0.0068
6	1	6	5	1	5	6575.1049	-0.0054
6	0	6	5	0	5	6590.0813	0.0008
3	3	0	2	2	0	6672.2074	-0.0002
5	3	2	4	3	1	6709.4569	0.0025
3	3	1	2	2	1	6729.5702	-0.0024
4	2	2	3	1	2	6906.8095	-0.0008
4	1	3	3	0	3	6918.0406	0.0045
5	2	3	4	2	2	6922.6435	-0.0028
6	2	5	5	2	4	7299.6114	-0.0029
4	2	3	3	1	3	7499.5476	-0.0040
6	1	5	5	1	4	7514.0536	0.0040
7	1	7	6	1	6	7608.0580	-0.0106
7	0	7	6	0	6	7613.4300	0.0109
6	3	4	5	3	3	7730.8206	-0.0023
6	4	3	5	4	2	7824.2755	0.0043
4	3	1	3	2	1	7877.1633	0.0036

Table S23. Measured frequencies and residuals (in MHz) of the rotational transitions of the parent species of *g*–*g*–*t*(II).

J'	*K'*$_{-1}$	*K'*$_{+1}$	*J''*	*K''*$_{-1}$	*K''*$_{+1}$	ν_{obs}	ν_{obs}-ν_{calc}
2	1	2	1	0	1	2692.6269	-0.0027
3	0	3	2	1	2	3549.2400	0.0034
2	1	1	1	0	1	3586.8389	0.0048
2	2	1	1	1	0	3794.2128	-0.0076
2	2	0	1	1	0	3945.7733	-0.0038
2	2	1	1	1	1	4092.2878	-0.0027
2	2	0	1	1	1	4243.8541	0.0069
4	2	2	3	3	1	4621.3863	-0.0086
4	0	4	3	1	3	4671.1577	0.0044
4	1	4	3	0	3	4709.5988	0.0010
3	2	2	2	1	1	4865.1145	0.0034
3	1	2	2	0	2	5418.7640	0.0074
3	2	1	2	1	1	5468.0947	0.0000
3	2	2	2	1	2	5759.3141	-0.0014
5	1	5	4	0	4	5765.0284	-0.0020
4	2	3	3	1	2	5796.9000	-0.0018
3	3	1	2	2	0	6043.6234	-0.0004
3	3	0	2	2	0	6099.7451	-0.0019
3	3	1	2	2	1	6195.1823	0.0019
3	3	0	2	2	1	6251.2966	-0.0071
5	2	3	4	3	2	6425.1764	0.0012
5	1	4	4	2	3	6454.3713	0.0107
5	2	4	4	1	3	6706.7519	-0.0030
6	0	6	5	1	5	6830.0210	-0.0046
6	1	6	5	0	5	6831.9447	-0.0030
4	2	2	3	1	2	7156.4584	-0.0018
4	3	2	3	2	1	7162.2700	0.0035
4	1	3	3	0	3	7369.1914	0.0023
4	3	1	3	2	1	7483.2771	-0.0068
4	2	3	3	1	3	7529.1730	0.0044
6	1	5	5	2	4	7610.1394	-0.0056
6	2	5	5	1	4	7688.0384	0.0067
4	3	2	3	2	2	7765.2498	-0.0003
7	0	7	6	1	6	7901.4247	0.0013
7	1	7	6	0	6	7901.8091	0.0007

Table S24. Cartesian coordinates of isomer *g-g+t*(I) from B3LYP-D3BJ/6-311++G(d,p) calculations.

	X	Y	Z
C	-3.095857	1.418577	0.372457
C	-1.839295	1.589080	-0.460342
H	-1.880342	0.949920	-1.352238
H	-1.747570	2.627917	-0.800882
O	-0.710073	1.241088	0.342373
H	0.096598	1.243501	-0.210199
H	-3.208672	0.380242	0.696253
H	-3.052334	2.049948	1.262912
H	-3.980700	1.695210	-0.206960
O	0.244912	-1.335692	0.736211
H	-0.303559	-0.530266	0.816098
C	-0.596006	-2.462056	0.486499
C	-1.380466	-2.337105	-0.814169
H	-0.704637	-2.191665	-1.660676
H	-2.069323	-1.488645	-0.777655
H	-1.970249	-3.241175	-0.993392
H	0.068744	-3.327934	0.449877
H	-1.279228	-2.609837	1.332392
O	1.578207	0.405583	-0.951334
H	1.322360	-0.411606	-0.480957
C	2.916126	0.755132	-0.583508
C	3.060438	1.025834	0.908708
H	2.400466	1.839265	1.220791
H	2.802769	0.137331	1.491359
H	4.090947	1.305733	1.147876
H	3.160842	1.647061	-1.164611
H	3.603732	-0.040063	-0.897280

Table S25. Cartesian coordinates of isomer *g+g+g−*(I) from B3LYP-D3BJ/6-311++G(d,p) calculations.

	X	Y	Z
C	-2.971613	1.692001	-0.260357
C	-1.759148	1.591583	0.654165
H	-1.605092	2.529150	1.192729
H	-1.910480	0.802264	1.402735
O	-0.552347	1.354534	-0.071963
H	-0.640607	0.516446	-0.567846
H	-2.834551	2.488766	-0.995039
H	-3.130776	0.753480	-0.800142
H	-3.874558	1.903968	0.320153
O	-0.071356	-1.227092	-0.989622
H	0.683619	-1.077768	-0.386063
C	-0.730330	-2.441841	-0.630141
C	-1.382886	-2.372254	0.745533
H	-0.637717	-2.167622	1.519150
H	-1.873335	-3.320536	0.985712
H	-2.136814	-1.581194	0.777807
H	-1.481603	-2.616561	-1.403769
H	-0.018522	-3.275561	-0.671164
O	1.657030	-0.058206	0.817823
H	0.988927	0.647886	0.714551
C	2.949126	0.473787	0.509804
C	3.043656	0.987349	-0.921289
H	2.825587	0.188824	-1.634946
H	2.330300	1.798374	-1.091082
H	4.049261	1.368019	-1.124565
H	3.654459	-0.343673	0.675349
H	3.197930	1.271212	1.220962

Table S26. Cartesian coordinates of isomer *g−tt*(I) from B3LYP-D3BJ/6-311++G(d,p) calculations.

	X	Y	Z
C	-2.492148	-2.359190	-0.244127
C	-1.109881	-2.072421	0.311542
H	-1.184032	-1.541391	1.269817
H	-0.571944	-3.011129	0.493752
O	-0.393248	-1.279422	-0.635863
H	0.456081	-0.994257	-0.244185
H	-3.045874	-1.430104	-0.405064
H	-2.418152	-2.881327	-1.200857
H	-3.062764	-2.982885	0.449155
O	-0.546972	1.483771	-0.867516
H	-0.774129	0.536309	-0.947446
C	-1.656080	2.195013	-0.317813
C	-2.038063	1.709489	1.075466
H	-1.186170	1.779486	1.756524
H	-2.370698	0.668093	1.050989
H	-2.855580	2.313846	1.480111
H	-1.351986	3.243611	-0.287025
H	-2.515478	2.123553	-0.996398
O	1.631064	0.308105	0.339820
H	1.021896	0.995321	0.006369
C	2.936930	0.509901	-0.207967
C	3.816428	-0.657455	0.197858
H	3.870067	-0.735873	1.286399
H	3.417156	-1.595821	-0.195844
H	4.829871	-0.525894	-0.190518
H	3.354844	1.451104	0.170426
H	2.878336	0.583973	-1.301121

Table S27. Cartesian coordinates of isomer *g*–*g*–*t*(II) from B3LYP-D3BJ/6-311++G(d,p) calculations.

	X	Y	Z
C	-0.024783	0.139377	0.022047
C	0.026402	-0.024685	1.536475
O	1.361839	-0.074033	2.040401
O	3.158690	-1.717043	0.726061
C	4.104768	-2.655463	1.242403
C	5.296028	-2.838942	0.312567
O	3.705909	0.963524	0.980028
C	3.667809	1.934495	-0.067667
C	4.965643	1.851280	-0.848611
H	1.805171	-0.870962	1.688531
H	2.843558	0.935046	1.438017
H	-0.450766	0.823588	2.032505
H	-0.516695	-0.928659	1.839014
H	0.481472	-0.692171	-0.475674
H	0.462315	1.069199	-0.283807
H	-1.061930	0.167030	-0.325909
H	3.543376	2.937049	0.360191
H	2.812963	1.742878	-0.729367
H	5.819036	2.036666	-0.192106
H	4.977883	2.593224	-1.651392
H	5.083137	0.860380	-1.295013
H	3.586154	-0.839471	0.655168
H	3.561164	-3.595549	1.360503
H	4.442838	-2.339705	2.237843
H	4.965612	-3.168521	-0.675451
H	5.986916	-3.585634	0.715652
H	5.845738	-1.900104	0.197029

S8. References

[1] D. Loru, M. A. Bermúdez, M. E. Sanz, *J. Chem. Phys.* **2016**, *145*, 074311.
[2] D. Loru, I. Peña, M. E. Sanz, *J. Mol. Spectrosc.* **2017**, *335*, 93–101.
[3] A. D. Becke, *J. Chem. Phys.* **1993**, *98*, 5648–5652.
[4] C. Lee, W. Yang, R. G. Parr, *Phys. Rev. B* **1988**, *37*, 785–789.
[5] S. Grimme, S. Ehrlich, L. Goerigk, *J. Comput. Chem.* **2011**, *32*, 1456–1465.
[6] E. R. Johnson, A. D. Becke, *J. Chem. Phys.* **2006**, *124*, 174104.
[7] M. J. Frisch, G. W. Trucks, H. B. Schlegel, G. E. Scuseria, M. A. Robb, J. R. Cheeseman, G. Scalmani, V. Barone, B. Mennucci, G. A. Petersson, H. Nakatsuji, M. Caricato, X. Li, H. P. Hratchian, A. F. Izmaylov, J. Bloino, G. Zheng, J. L. Sonnenberg, M. Hada, M. Ehara, K. Toyota, R. Fukuda, J. Hasegawa, M. Ishida, T. Nakajima, Y. Honda, O. Kitao, H. Nakai, T. Vreven, J. Montgomery, J. A., J. E. Peralta, F. Ogliaro, M. Bearpark, J. J. Heyd, E. Brothers, K. N. Kudin, V. N. Staroverov, R. Kobayashi, J. Normand, K. Raghavachari, A. Rendell, J. C. Burant, S. S. Iyengar, J. Tomasi, M. Cossi, N. Rega, J. M. Millam, M. Klene, J. E. Knox, J. B. Cross, V. Bakken, C. Adamo, J. Jaramillo, R. Gomperts, R. E. Stratmann, O. Yazyev, A. J. Austin, R. Cammi, C. Pomelli, J. W. Ochterski, R. L. Martin, K. Morokuma, V. G. Zakrzewski, G. A. Voth, P. Salvador, J. J. Dannenberg, S. Dapprich, A. D. Daniels, Ö. Farkas, J. B. Foresman, J. V. Ortiz, J. Cioslowski, D. J. Fox, *Gaussian 09, Revision E. 01; Gaussian*, **2009**.
[8] P. Pracht, F. Bohle, S. Grimme, *Phys. Chem. Chem. Phys.* **2020**, *22*, 7169–7192.
[9] A. Malloum, A. Malloum, J. J. Fifen, J. Conradie, *Phys. Chem. Chem. Phys.* **2020**, *22*, 13201–13213.
[10] S. F. Boys, F. Bernardi, *Mol. Phys.* **1970**, *19*, 553–566.
[11] L. Uribe, F. Lazzari, S. Di Grande, L. Crisci, M. Mendolicchio, V. Barone, *J. Chem. Phys.* **2024**, *161*, 14307.
[12] F. Xie, M. Mendolicchio, W. Omarouayache, S. I. Murugachandran, J. Lei, Q. Gou, M. E. Sanz, V. Barone, M. Schnell, *Angew. Chemie Int. Ed.* **2024**, *63*, e202408622.
[13] S. Grimme, F. Bohle, A. Hansen, P. Pracht, S. Spicher, M. Stahn, *J. Phys. Chem. A* **2021**, *125*, 4039–4054.
[14] S. Grimme, J. G. Brandenburg, C. Bannwarth, A. Hansen, *J. Chem. Phys.* **2015**, *143*, 054107.
[15] J. G. Brandenburg, E. Caldeweyher, S. Grimme, *Phys. Chem. Chem. Phys.* **2016**, *18*, 15519–15523.
[16] A. V. Marenich, C. J. Cramer, D. G. Truhlar, *J. Phys. Chem. B* **2009**, *113*, 6378–6396.
[17] R. S. Ruoff, T. D. Klots, T. Emilsson, H. S. Gutowsky, *J. Chem. Phys.* **1990**, *93*, 3142–3150.
[18] G. M. Florio, R. A. Christie, K. D. Jordan, T. S. Zwier, *J. Am. Chem. Soc.* **2002**, *124*, 10236–10247.
[19] O. Mó, M. Yáñez, J. Elguero, *J. Chem. Phys.* **1997**, *107*, 3592–3601.
[20] N. A. Seifert, A. L. Steber, J. L. Neill, C. Pérez, D. P. Zaleski, B. H. Pate, A. Lesarri, *Phys. Chem. Chem. Phys.* **2013**, *15*, 11468–11477.
[21] J. Thomas, N. A. Seifert, W. Jäger, Y. Xu, *Angew. Chemie - Int. Ed.* **2017**, *56*, 6289–6293.

S9. Author Contributions

Conceptualization, M.E.S.; formal analysis, S.I.M, I.P., A.M.L., M.Y. and M.E.S.; investigation, S.I.M, I.P., A.M.L., M.Y. and M.E.S.; data curation, S.I.M, I.P., A.M.L., M.Y. and M.E.S..; writing—original draft preparation, S.I M.; writing—review and editing, S.I.M, I.P., A.M.L., M.Y. and M.E.S.; project administration, A.M.L., M.Y. and M.E.S.; funding acquisition, A.M.L., M. Y. and M.E.S. All authors have read and agreed to the published version of the manuscript.
